# The selfish yeast plasmid utilizes the condensin complex and condensed chromatin for faithful partitioning

**DOI:** 10.1371/journal.pgen.1009660

**Published:** 2021-07-16

**Authors:** Deepanshu Kumar, Hemant Kumar Prajapati, Anjali Mahilkar, Chien-Hui Ma, Priyanka Mittal, Makkuni Jayaram, Santanu K. Ghosh

**Affiliations:** 1 Department of Biosciences and Bioengineering, Indian Institute of Technology, Bombay, Powai, Mumbai, India; 2 Department of Chemical Engineering, Indian Institute of Technology, Bombay, Powai, Mumbai, India; 3 Section of Molecular Genetics and Microbiology, University of Texas, Austin, Texas, United States of America; Fred Hutchinson Cancer Research Center, UNITED STATES

## Abstract

Equipartitioning by chromosome association and copy number correction by DNA amplification are at the heart of the evolutionary success of the selfish yeast 2-micron plasmid. The present analysis reveals frequent plasmid presence near telomeres (*TEL*s) and centromeres (*CEN*s) in mitotic cells, with a preference towards the former. Inactivation of Cdc14 causes plasmid missegregation, which is correlated to the non-disjunction of *TEL*s (and of rDNA) under this condition. Induced missegregation of chromosome XII, one of the largest yeast chromosomes which harbors the rDNA array and is highly dependent on the condensin complex for proper disjunction, increases 2-micron plasmid missegregation. This is not the case when chromosome III, one of the smallest chromosomes, is forced to missegregate. Plasmid stability decreases when the condensin subunit Brn1 is inactivated. Brn1 is recruited to the plasmid partitioning locus (*STB*) with the assistance of the plasmid-coded partitioning proteins Rep1 and Rep2. Furthermore, in a dihybrid assay, Brn1 interacts with Rep1-Rep2. Taken together, these findings support a role for condensin and/or condensed chromatin in 2-micron plasmid propagation. They suggest that condensed chromosome loci are among favored sites utilized by the plasmid for its chromosome-associated segregation. By homing to condensed/quiescent chromosome locales, and not over-perturbing genome homeostasis, the plasmid may minimize fitness conflicts with its host. Analogous persistence strategies may be utilized by other extrachromosomal selfish genomes, for example, episomes of mammalian viruses that hitchhike on host chromosomes for their stable maintenance.

## Author summary

The multi-copy 2-micron plasmid, a nearly ubiquitous resident in budding yeast nuclei, has almost the same stability as its host’s chromosomes. The plasmid partitioning system, together with host-coded factors, apparently positions plasmid molecules at chromosome sites, enabling their chromosome-coupled segregation. Here, we demonstrate that plasmid association with chromosomes is not random. In vegetative yeast cells, telomeres and centromeres are among preferred plasmid localization sites, with an obvious bias favoring the former. Increased plasmid missegregation under forced non-disjunction of condensed chromosome loci such as telomeres and rDNA, or under induced missegregation of chromosome XII harboring the rDNA array, suggest a possible role for the condensin complex in plasmid segregation. This notion is supported by the interaction of the condensin component Brn1 with the plasmid partitioning proteins, its recruitment to the plasmid partitioning locus with the assistance of these proteins, and increased plasmid loss when Brn1 is inactivated. Our collective findings suggest that the plasmid targets condensed/heterochromatin-like chromosome regions for its chromosome association. The plasmid may thus minimize perturbations of genome functions, and optimize its evolutionary fitness without compromising that of the host. Analogous strategies may be utilized by selfish DNA elements in general, including the episomes of certain mammalian viruses.

## Introduction

Eukaryotes, in contrast to prokaryotes, rarely harbor stably propagating extra-chromosomal DNA elements. However, circular plasmids have been identified in budding yeasts and in the slime mold *Dictyostelium* [1, 2]. In addition, papilloma and gammaherpes viruses exist as episomes in infected mammalian cells during long periods of latency [3–5]. Eukaryotic nuclei also contain extra-chromosomal circular DNA molecules, called eccDNA that are excised from chromosomes [6–8]. Their formation, often associated with DNA replication/repair events, may be important in the evolutionary sizing and shaping of genomes. A subset of these circles has been associated with human diseases [9, 10].

The multi-copy 2-micron plasmid of *Saccharomyces cerevisiae*, present as 40–60 molecules per haploid genome content, is the most well characterized among yeast DNA plasmids [11–14]. Current evidence suggests that plasmid molecules are organized into clusters of 3–5 foci that act as units of segregation during cell division [15–17]. The plasmid genome has a bi-partite functional organization, namely, a replication-partitioning module and a copy number amplification module. Plasmid molecules duplicated by the host replication machinery [18] are segregated equally (or nearly so) to daughter cells by the plasmid-coded Rep1 and Rep2 proteins acting in conjunction with the *cis*-acting *STB* locus [12–14]. The Flp site-specific recombination/amplification system housed by the plasmid compensates for copy number reduction resulting from rare missegregation events. Iterative copying of the plasmid template is triggered by a Flp-mediated recombination event coupled to bidirectional plasmid replication [19–21]. Mutually reinforcing regulatory mechanisms harbored by the plasmid [22–25] and imposed by the host [26–28] protect against any undue drop or runaway increase in plasmid population. Equal segregation and copy number optimization account for the evolutionary persistence of the 2-micron plasmid as a selfish DNA element that neither enhances nor significantly diminishes host fitness [29, 30].

The current model for 2-micron plasmid propagation, supported by several lines of circumstantial evidence, posits plasmid segregation in physical association with chromosomes (hitchhiking) [16, 31, 32]. Furthermore, plasmid sisters formed by replication of a single copy *STB*-reporter segregate in a one-to-one fashion, suggesting their association with sister chromatids [17, 32]. This chromosome-like segregation is orchestrated by the Rep-*STB* system with the assistance of several host factors that also promote *CEN*-mediated chromosome segregation [17, 31, 33–40]. It has been speculated that the unusually short, and genetically defined, point-*CEN* of budding yeast chromosomes and the plasmid-*STB* have diverged from a common ancestor that once directed both plasmid and chromosome segregation [37, 41]. Extrapolation of the single-copy plasmid behavior to the native multi-copy plasmid foci suggests a high-order organization within each focus for coordinating plasmid replication with symmetric attachment of the replicated copies to sister chromatids.

Fluorescence-tagged *STB*-reporter plasmids have revealed their tendency to localize near *CEN*s and spindle pole bodies in mitotic cells [15], and at or near *TEL*s in meiotic cells [42]. Plasmid-*TEL* association is dependent on the meiosis-specific bouquet proteins Ndj1 and Csm1 [43–47]. The role of the mitotic spindle, the spindle-associated Kip1motor and the microtubule plus end binding Bik1 and Bim1 proteins in 2-micron plasmid segregation during mitosis [34, 35, 40] suggests spindle-mediated recruitment of the plasmid to chromosome sites in the vicinity of *CEN*s. The association of the *CEN*-specific histone H3 variant Cse4 with *STB* [38], which is additionally detected at *CEN*-like regions (CLRs) [48], would also be consistent with the proximity between plasmid and *CEN*s. The theme of plasmid segregation by attaching to chromosomes appears to be conserved during both mitosis and meiosis with variations in the mode of plasmid-chromosome association and in host factors responsible for such association as necessitated by the two distinct cell cycle programs.

The apparent differential plasmid localization on chromosomes in mitotic versus meiotic cells may, in principle, be reconciled by structural or organizational features common to *CEN*s and *TEL*s, and perhaps shared by other plasmid tethering sites on chromosomes. To address more definitively the still unresolved questions regarding chromosome locations utilized by the 2-micron plasmid for its association with chromosomes, we have now mapped the positions of a single-copy *STB*-reporter plasmid with respect to *CEN V* and *TEL V* (also *TEL V* and *TEL VII*) simultaneously. We find that the plasmid is located near *CEN* or *TEL* in ≥ 65% of the mitotic cells (≤ 0.5 μm), with a clear *TEL* preference over *CEN*. Interestingly, 2-micron plasmid segregation is impaired when the disjunction of strongly condensed loci such as *TEL*s or rDNA is blocked by Cdc14 inactivation. Consistent with this observation, induced missegregation of a long and highly condensin-dependent chromosome (Chr XII) has a stronger negative effect on equal plasmid segregation than the missegregation of a short and less condensin-dependent chromosome (Chr III). Genetic assays and chromatin immunoprecipitation (ChIP) analyses reveal the interaction of the condensin subunit Brn1 with the Rep-*STB* system, suggesting that condensin is an authentic plasmid partitioning factor appropriated from the host. Condensin requirement for plasmid segregation may be mandated by the need for multiple plasmid molecules within a focus to be condensed in order for them to co-segregate with a chromosome as one physical entity. Preferential localization at chromosome loci where condensin is enriched may reflect a plasmid strategy to satisfy this requirement. Alternatively, condensin acquisition by the clustered plasmid molecules, and presumably their condensation, may facilitate the tethering of plasmid foci to condensed chromosome locales. By associating with condensed, quiescent chromatin such as *TEL*s, the plasmid may minimize potential deleterious effects on chromosome function and on the host’s fitness.

## Results

### Strategy for mapping plasmid positions with respect to *CEN*s, *TEL*s and the spindle pole body at different stages of mitosis

Current evidence suggests that the 2-micron plasmid tends to localize preferentially near the *CEN* cluster and the spindle pole body in mitotic cells [35], but migrates towards the nuclear periphery as diploid cells enter the meiotic program [42, 49]. These localizations were carried out primarily using fluorescence-tagged multi-copy *STB*-reporter plasmids [35, 42], and a nearly single-copy reporter plasmid in one instance [40]. Visualization of the native 2-micron plasmid in mitotic cells using FISH also reveals a significantly higher number of plasmid foci unassociated with the nuclear membrane than those localizing with it [50]. Meiotic plasmid localization at the nuclear periphery increases from early meiosis to the pachytene stage, and requires the bouquet proteins Ndj1 and Csm4 [42]. In pachytene chromosome spreads, over half the plasmid foci are situated on chromosomes, predominantly at or near *TEL*s [42]. The 2-micron plasmid may exploit chromatin features characteristic of *CEN*s or *TEL*s, or regions proximal to them, for its chromosome localization. Plasmid presence distal to these loci may be accounted for by similar chromatin features present at a subset of other chromosome regions or by alternative chromatin features recognized by the Rep-*STB* system. Differential plasmid localization with respect to the nuclear periphery during mitosis and meiosis may reflect changes in chromatin organization and/or altered spatial arrangements of chromosome locales between mitotic and meiotic cells.

In order to gain further insights into the chromosome-associated segregation of the 2-micron plasmid, we sought to map plasmid positions in mitotic cells at higher resolution by simultaneously fluorescence-tagging Chr V at *CEN* and the right arm *TEL*. Furthermore, to avoid any imprecision posed by multiple plasmid foci, we employed a previously described *STB*-reporter plasmid (pSG1) present at one copy (and as a single focus) in ≥ 80% of the cells [17]. The segregation behavior of this plasmid is quite similar to that of an *STB*-reporter plasmid excised from a chromosome by site-specific recombination at G1, and therefore has a copy number of exactly one prior to DNA replication [16]. A unique advantage of pSG1, which also contains a *GAL* promoter-regulated *CEN*, is that it can be induced to behave as a *CEN*-plasmid, *STB*-plasmid or an *ARS*-plasmid (lacking partitioning activity) under appropriate experimental conditions (S1 Fig). When cells harboring pSG1 are grown in medium with glucose as the carbon source (to repress the *GAL* promoter), it is referred to as pSG1-*CEN* to indicate an active *CEN*. The *CEN*-mediated partitioning function of pSG1-*CEN* is not affected by the presence or absence of the native 2-micron plasmid in the host cells ([Cir^+^] or [Cir^0^], respectively). When present in [Cir^+^] cells, and the carbon source is galactose, *CEN* is inactivated (by high level transcription from the *GAL* promoter), and the plasmid is referred to as pSG1*-STB* to denote its segregation by a functional Rep-*STB* partitioning system. When placed in [Cir^0^] cells with galactose as the carbon source, the absence of the Rep proteins and lack of *CEN* function make the plasmid behave as pSG1-*ARS* in segregation (S1 Fig).

We distinguished *CEN V* and *TEL V* in cells expressing TetR-GFP by their distinct fluorescence intensities conferred by [TetO]_224_ and [TetO]_448_ arrays inserted at *CEN V* and *TEL V* loci, respectively [51, 52] (S2 Fig). We verified that the two loci could be identified unambiguously at various stages of mitosis by marking their positions relative to the spindle pole body (SPB) tagged with Spc42-mCherry (S2 Fig). In *S*. *cerevisiae*, *CEN*s remain congressed as a single cluster in close proximity to SPB during interphase as well as mitosis, while the rosette of three to six *TEL* clusters is positioned at the nuclear periphery distal to SPB in a Rabl-like pattern [53–59]. Consistent with this spatial organization, we observed partial overlap or close proximity of SPB to the fainter of the two GFP foci (*CEN V*), with the brighter GFP focus *(TEL V)* located away from it (S2 Fig). These three nuclear landmarks provided a frame of reference for mapping the spatial positions of reporter plasmids.

### Mitotic localization of pSG1-*CEN* and pSG1-*STB* with respect to *CEN V* and *TEL V*

In order to follow plasmid localization in the nucleus, we introduced pSG1 into isogenic [Cir^+^] and [Cir^0^] strains containing the marked *CEN V*, *TEL V* and SPB and expressing cyan-LacI. Plasmid foci were visualized by repressor interaction with the plasmid-borne [LacO]_256_ array [60]. As already noted, by using [Cir^+^] and [Cir^0^] backgrounds and manipulating the carbon source, we could induce pSG1 to behave as pSG1-*CEN*, pSG1-*STB* or pSG1-*ARS* (S1 Fig, [17]).

**Fig 1:**
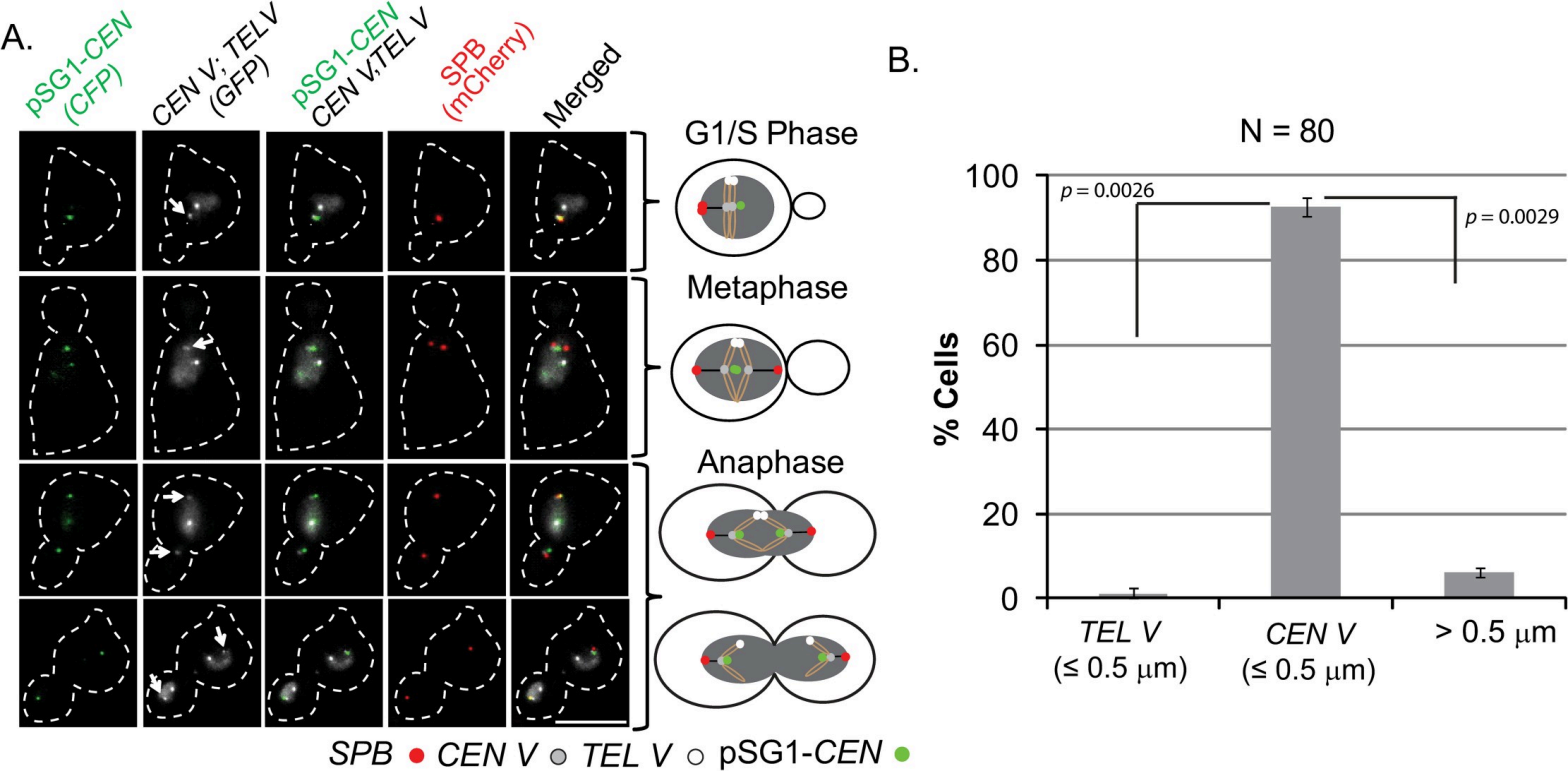
Localization of pSG1-*CEN* plasmid with respect to *CEN V* and *TEL V* during different stages of the cell cycle. **A**. The positions of pSG1-*CEN*, along with those of *CEN V* (indicated by arrows) and *TEL V*, were followed in [Cir^+^] cells grown in glucose (to keep the *CEN* harbored by the plasmid active). Representative images from fixed cells are shown in rows at the left, and summarized in the schematic diagrams at the right. G1/S in this Figure (also in Figs 2 and 3) refers to cells released from G1 arrest and in the early stage of bud emergence. Fluorescence tags: pSG1-*CEN*, [LacO]_256_-[CFP-LacI]; *CEN V*, [TetO]_224_-[TetR-GFP]; *TEL V*, [TetO]_448_-[TetR-GFP]; SPB, (Spc42-mCherry). **B**. pSG1-*CEN* locations proximal to a reference locus (≤ 0.5 μm) or distal from either of the two reference loci (> 0.5 μm) are shown in the plots. The *p* values were obtained by the two-tailed Student’s t-test. Bar, 5 μm.

**Fig 2:**
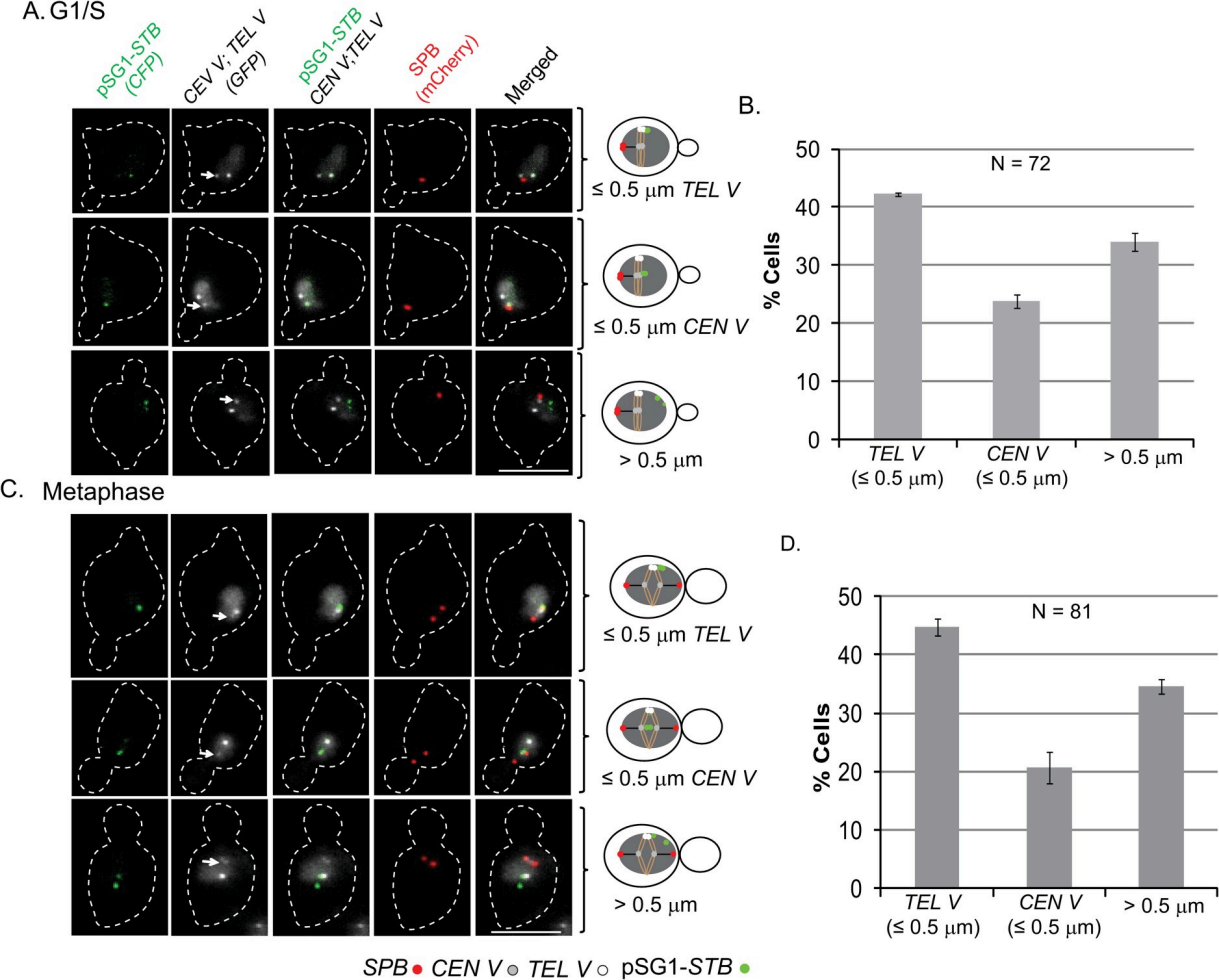
Preferred *TEL*-proximal localization of pSG1-*STB*. The localization of pSG1-*STB* ([Cir^+^]; galactose) with respect to *CEN V* and *TEL V* was scored in fixed cells at the G1/S (**A**) and metaphase (**C**) stages of the cell cycle. The types of plasmid localization with respect to reference loci represented by the rows of images at the left are codified in the corresponding schematic diagrams placed to the right of each row. Fluorescence tags: pSG1-*STB*, [LacO]_256_-[CFP-LacI]; *CEN V*, [TetO]_224_-[TetR-GFP]; *TEL V*, [TetO]_448_-[TetR-GFP]; SPB, (Spc42-mCherry). The histogram plots in (**B)** and (**D**) are based on the data from (**A**) and (**C**), respectively. For those cells that showed two resolved plasmid foci, the ≤ 0.5 μm criterion for proximity applied to both. Even when one plasmid focus failed this distance limit, the classification was > 0.5 μm. Bar, 5 μm.

The localization of pSG1-*CEN* ([Cir^+^]; glucose) at different stages of the mitotic cell cycle (Fig 1A and 1B) at or close to SPB (≤ 0.5 μm) was akin to that of chromosome *CEN*s (S2 Fig), as expected. The spatial distribution of pSG1-*STB* ([Cir^+^]; galactose), however, was different (Figs 2 and S3). In G1/S (Fig 2A and 2B) and in metaphase (Fig 2C and 2D) cells, 40–45% of the cyan foci were proximal to the *TEL V* (≤ 0.5 μm), 20–25% were close to *CEN V* (≤ 0.5 μm) and the rest showed no spatial linkage to either *CEN V* or *TEL V* (> 0.5 μm) (Fig 2A–2D). As the average copy number of pSG1 was only approximately one [17], not every cell in the population contained a single plasmid molecule prior to replication. Once replicated, pSG1-*STB* sisters would remain paired in most cells until anaphase onset, consistent with their bridging by the cohesin complex [16, 31, 39] and/or their symmetric attachment to cohesin-paired sister chromatids [32]. In 60–65% of metaphase cells, pSG1-*STB* was seen as a single focus of coalesced sisters. The cells scored for the distance estimates contained predominantly one pSG1-*STB* focus, but included a small number displaying two plasmid foci. A cell belonging to the latter subset was placed in the > 0.5 μm category even when only one focus showed this localization pattern (Fig 2C, row 3). All cells with more than two plasmid foci, indicating a copy number > 1, were excluded from the analysis.

As a more stringent test of the apparent tendency of pSG1-*STB* to remain *TEL*-proximal, we observed its localization in a selected subset of anaphase cells in which the *CEN V*s were well separated from each other, and from the unsegregated *TEL V*s that remained at the spindle mid-zone (S3A Fig). In this population, the fraction of cells showing pSG1-*STB* proximity to *TEL V* was ~45%; *CEN V* proximity was seen in ~15% (S3B Fig). Here too, assignment of a cell to the ≤ 0. 5 μm class required both copies of the plasmid to be compliant to this criterion.

Finally, we verified the mapping of plasmid positions by determining the three-dimensional disposition of pSG1 with respect to *CEN V* and *TEL V* in live cells. The dot plots of distances in S4 Fig were derived by defining the centroids of the plasmid and chromosome fluorescent foci from imaging a series of nuclear stacks (S4A Fig). The distribution of pSG1-*STB* showed a discernible bias towards *TEL* proximity (S4B Fig), while the expected *CEN*-proximal localization of pSG1-*CEN* was also evident (S4C Fig).

Taken together, the results from Figs 2 and S3 suggest that the 2-micron plasmid occupies *TEL*/*CEN* vicinities in well over half the cell population with a strong bias for *TEL*. At all stages of the cell cycle, at least a third of the cells showed no association of pSG1-*STB* with *CEN V* or *TEL V* (> 0.5 μm in Figs 2 and S3). In our assay, a plasmid mapped distal to *TEL V* may still be associated with a *TEL* cluster unmarked by the fluorescence tag. Conversely, due to *TEL* commingling, *TEL V* proximity of a plasmid represents its association with any one of the *TEL*s present in the *TEL V* containing cluster. Localization distal to *TEL*/*CEN* would also be consistent with plasmid presence at other tethering sites distributed along chromosome arms.

### Localization of pSG1-*ARS*

To verify the role of the Rep-*STB* system in directing plasmids to preferred chromosome locales, we examined how the *CEN V*-*TEL V*-based map positions are affected for a control *ARS*-plasmid.

**Fig 3:**
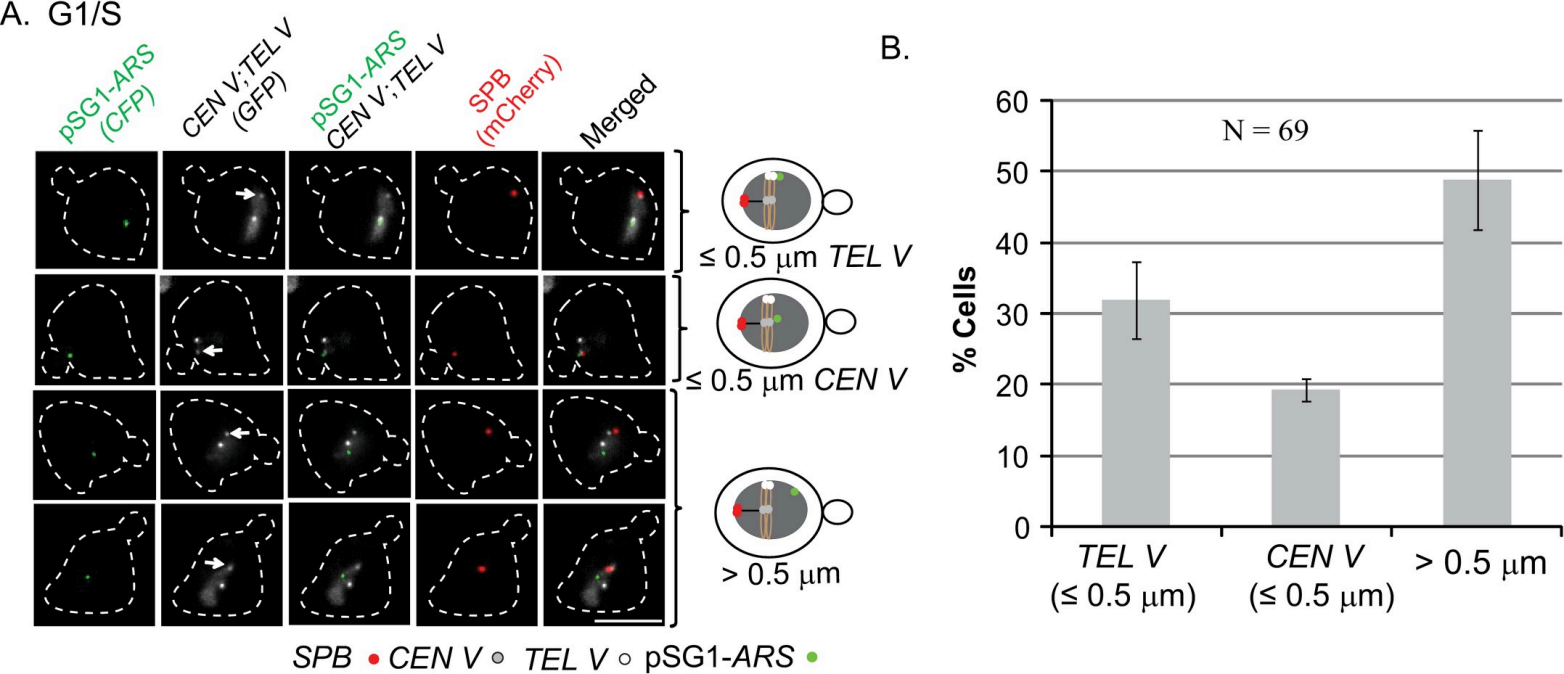
Localization of pSG1-*ARS* in the nuclei of mitotic cells at the G1/S stage. **A**. Cells ([Cir^0^]; galactose) were observed at G1/S. The cell images (left) and the corresponding schematic diagrams (right) represent the three localization patterns of pSG1-*ARS*. Fluorescence tags: pSG1-*ARS*, [LacO]_256_-[CFP-LacI]; *CEN V*, [TetO]_224_-[TetR-GFP]; *TEL V*, [TetO]_448_-[TetR-GFP]; SPB, (Spc42-mCherry). **B**. The relative abundance of each pattern is shown in the histogram plot. Bar, 5 μm.

For the pSG1-*ARS* plasmid, the proximal (≤ 0.5 μm from *CEN V* or *TEL V*) and distal (> 0.5 μm from *CEN V* and *TEL V*) locations were roughly evenly divided among G1/S cells (Fig 3). There was no significant statistical difference (Chi-square test; *p* = 0.0666, α = 0.05) from the localization of pSG1-*STB* (~65% *CEN V*/*TEL V*-proximal; Fig 2A). Viewed in isolation, this finding might suggest that the Rep-*STB* system does not direct plasmid localization in the nucleus. Conversely, localization has no role in plasmid segregation. However, this interpretation would seem untenable in light of a variety of prior observations summarized below. Furthermore, pSG1-*ARS* positions mapped by live cell imaging were essentially random, spread over a wide range from *CEN V* or *TEL V* (S4D Fig).

Unlike *STB*-plasmids, *ARS*-plasmids do not physically associate with chromosomes [12, 31]. A previous study showed pSG1-*CEN*, pSG1-*STB* and pSG1-*ARS* to be present in ≥ 90%, ~80% and <20%, respectively, of chromosome spreads prepared from G1 cells [32]. Furthermore, Rep1, Rep2 and pSG1-*STB* form coalesced foci in these spreads [32]. Multi-copy *STB*-plasmids also associate with mitotic and meiotic chromosome spreads in a Rep1-Rep2-dependent fashion [31, 42]. Higher resolution of the meiotic spreads revealed plasmid foci, a considerable fraction of which is present on chromosomes at or near their tips, to be colocalized with a subset of the Rep protein foci [42]. The evidence for the functional relevance of chromosome association to *STB*-plasmid segregation is also compelling [15, 16, 31, 32, 42] (see also ‘Discussion’).

Given the striking differences between *STB*- and *ARS*-plasmids in physical chromosome association and chromosome-associated segregation, the localization of pSG1-*ARS* seen in Fig 3 is best explained as random plasmid-chromosome encounters within the crowded nucleus. *ARS*-plasmid proximity or remoteness to *CEN*-*TEL* in the Fig 3 plots is qualitatively consistent with the tightly congressed single *CEN* cluster (~20% proximal to *CEN V*), the less tightly compacted 3–6 *TEL* clusters (~30% proximal to *TEL V*) and the remainder of the chromosome mass (50% distal to *CEN V*-*TEL V*).

### Association of the 2-micron plasmid with *TEL VII*

Unlike *CEN*s, which are congressed into one tightly knit cluster in mitotic cells, *TEL*s form a rosette of 3–6 clusters [53, 54]. The premise of our assays (Figs 2 and S3) is that *TEL V* is equally likely to associate with any of these clusters. In order to probe potential variations among *TEL*s in plasmid association, we assayed plasmid localization with respect to two *TEL*s, *TEL V* and *TEL VII* (on the right arm of Chr VII), differentially tagged by [TetO]_448_-[TetR-GFP] and [TetO]_~50_-[TetR-GFP] fluorescence, respectively.

In the experimental strain, *TEL V* was distinguished from *TEL VII* by the difference in their fluorescence intensities. The nearly same frequency of pSG1 association with *TEL V* and *TEL VII* (35–40%; S5A Fig) suggests that the Rep-*STB* system may recognize shared high-order chromatin organization and/or epigenetic features present at or near *TEL*s. The overlap (< 0.5 μm) between *TEL V* and *TEL VII* (~20%; S5B Fig) indicates their presence in a single *TEL* cluster or two closely spaced clusters. Proximity is somewhat broadly defined in our assays because of the resolution limits of fluorescence microscopy and the cut-off distance of 0.5 μm employed.

In sum, the plasmid localization data would be consistent with *TEL*/*CEN* association constituting a major segregation route for the 2-micron plasmid in the chromosome hitchhiking model. Preferential plasmid segregation as a *TEL* appendage observed during meiosis [42] appears to hold for mitosis as well, a fact that was not appreciated from prior published results. While Rep1 and Rep2 proteins are essential for mediating plasmid-chromosome association in both mitosis and meiosis, the host proteins involved in plasmid linkage to *TEL*s are likely different between the two cell cycle programs [42].

### Blocking segregation of condensed chromosome loci causes *STB*-plasmid non-disjunction

In the budding yeast, *TEL*-proximal regions are rich in the condensin complex, and are organized as condensed, transcriptionally quiescent heterochromatin [61]. *CEN*s act as *cis*-acting mediators to promote recruitment of condensin, cohesin and associated signaling molecules to pericentric regions, which have characteristic chromatin composition and organization [62–65]. Genome-wide mapping studies reveal quantitative differences in condensin occupancy along chromosome arms, as well as cell cycle dependent modulations in occupancy at individual loci [61]. In mitotic yeast cells, the rDNA gene cluster is also prominent in condensin enrichment. The non-random localization of an *STB*-plasmid on chromosomes, favoring the vicinity of *TEL*s and *CEN*s (Figs 2 and S3–S5), prompted us to examine a potential role for condensin and/or condensed chromatin in 2-micron plasmid segregation.

**Fig 4:**
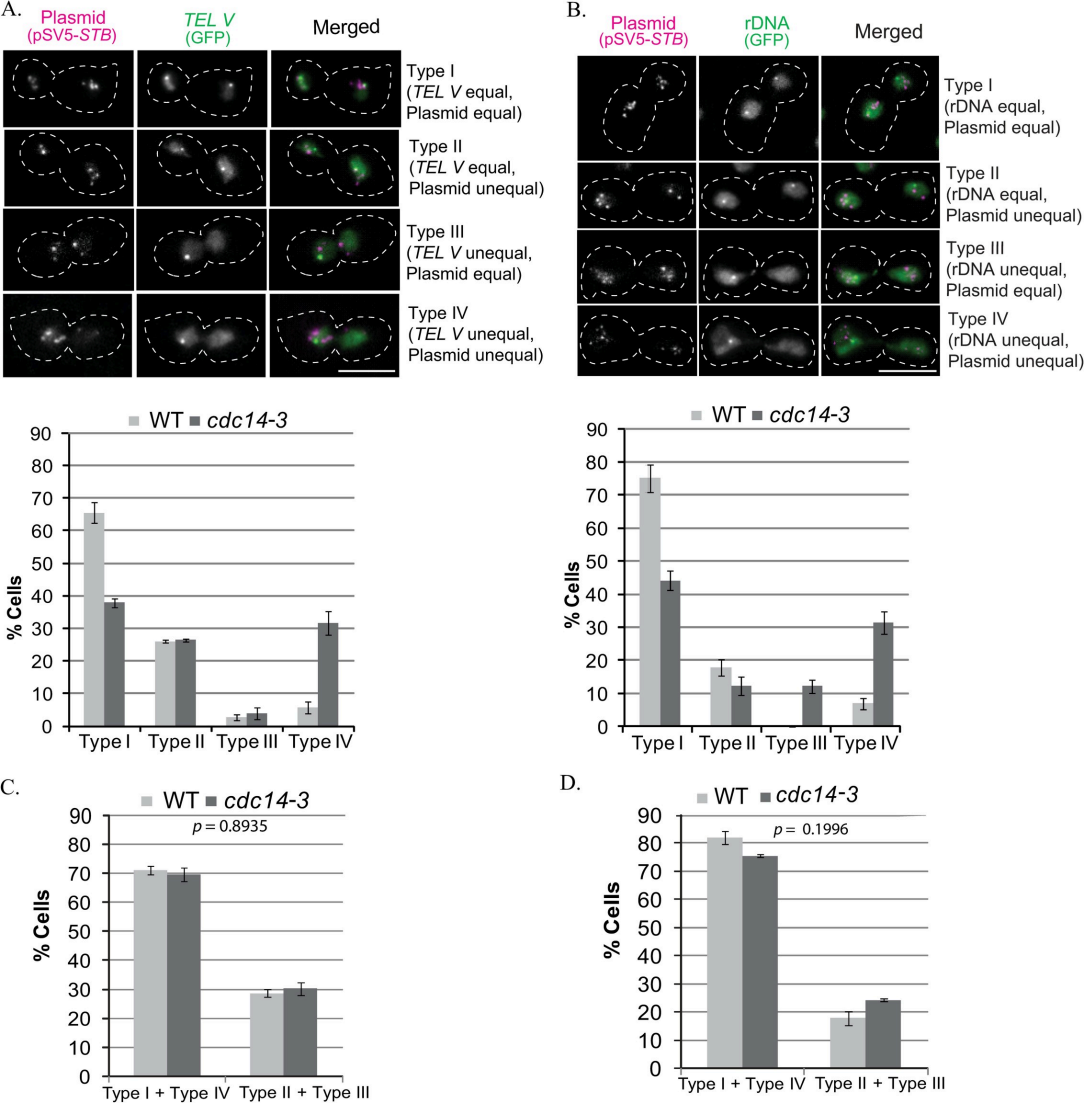
2-micron plasmid segregation when Cdc14 inactivation causes missegregation of *TEL*s or rDNA. **A**, **B**. The wild type and *cdc14-3* strains (otherwise isogenic) were arrested in G1 with α-factor at 23°C, and released from arrest at 33°C. Representative images of the four cell types analyzed are shown in the top panels (pSV5-*STB*, *TELV*) in (**A**); (pSV5-*STB*, rDNA) in (**B**). Fluorescence tags: pSV5-*STB*, [LacO]_256_-[CFP-LacI]; *TEL V*, [TetO]_448_-[TetR-GFP]; rDNA, [TetO]_448_-[TetR-GFP]. The histogram plots for the quantitative estimates of the cell types are shown below the respective cell type panels. The differences in plasmid segregation correlated with *TEL V* or rDNA segregation (Type I + Type IV) and uncorrelated with the segregation of these loci (Type II + Type III) are underscored in the plots at the bottom (**C**, **D**). The data were obtained from more than 100 and 160 cells in each assay set for the wild type and *cdc14-3* cells, respectively. Statistical significance was estimated by Fisher’s exact test. Bar, 5 μm.

*TEL*s and rDNA differ subtly from chromosomes as a whole in their segregation mechanism. In addition to early anaphase cleavage of cohesin that holds together sister chromatids in pairs, these loci require a second ‘late’ condensin-dependent step to complete unlinking and segregation [66]. The extra condensin recruitment is mediated by Cdc14 phosphatase, which is released from its sequestered state at the onset of anaphase by the FEAR (Fourteen Early Anaphase Release) network [67, 68]. Upon inactivation of Cdc14, rDNA and *TEL*s are delayed in disjunction relative to the rest of the chromosomes [69]. If *TEL*-proximity of the 2-micron plasmid is functionally relevant to its partitioning, perhaps by way of condensed chromatin, lack of Cdc14 may be expected to increase plasmid missegregation. We tested this prediction using a multi-copy *STB*-reporter plasmid pSV5-*STB* (Fig 4) that we had utilized in prior plasmid segregation assays [31]. The control reporter plasmids for this analysis were derivatives of pSV5 in which *STB* was either deleted (pSV6-*ARS*) or replaced by *CEN* (pSV7-*CEN*) (S6 and S7 Figs).

The pSV5 plasmid, fluorescence-tagged by [LacO]-[CFP-LacI] interaction, is organized into 2–5 foci per nucleus. Earlier experiments suggest that each plasmid focus is an independent unit in segregation [15]. As the number of plasmid molecules in individual foci is unknown, and occasionally foci tend to overlap, segregation estimates from counting foci numbers in mother and daughter nuclei are only semi-quantitative. Nevertheless, there is good agreement between data obtained from multi-copy and single-copy plasmid reporters in segregation assays [15–17].

In the *cdc14-3* (Ts) strain at 33°C (non-permissive), a significant fraction of the cells showed non-disjunction of *TEL*s marked by [*TEL V*-TetO]-[TetR-GFP] (~35%; Type III + Type IV, Fig 4A) or the r-DNA locus marked by [rDNA-TetO]-[TetR-GFP] (~40%; Type III + Type IV, Fig 4B), as expected from prior studies [70]. In the wild type strain, these loci segregated normally in ≥ 90% of the cells (Type I + Type II, Fig 4A and 4B). The corresponding equal segregation of ~70% for pSV5-*STB* (Type I + Type III, Fig 4A and 4B), which is consistent with previous results for multi-copy and single-copy reporter plasmids [15, 17], dropped to ~50% in the *cdc14* mutant (Type I + Type III, Fig 4A and 4B). The decrease in equal segregation due to Cdc14 inactivation, when normalized for the differences in the wild type host, was comparable between the *STB*-plasmid (~30%; Type I + Type III) and *TEL V* or rDNA (35–40%; Type I + Type II). Significantly, pSV5-*STB* and *TEL V* or rDNA were well correlated in their segregation, equal as well as unequal, in the wild type and mutant strains (Type I + Type IV >> Type II + Type III; Fig 4C and 4D). Note also that the fraction of Type II + Type III cells, representing plasmid segregation not correlated to *TELV* or rDNA segregation, was essentially unchanged by the *cdc14* mutation. Or, the decrease in correlated equal segregation (Type I) in the mutant was matched nearly quantitatively by the increase in correlated missegregation (Type IV), as revealed in the near equality of Type I + Type IV between wild type and *cdc14* in the plots in Fig 4C and 4D. In the majority of cells in which plasmid missegregation was coupled to the block in *TEL V* or rDNA segregation (Type IV, Fig 4A and 4B), the entire cluster of pSV5-*STB* foci, or a majority of the foci, was trapped in the mother or at the bud neck.

In contrast to pSV5-*STB*, pSV6-*ARS* [31], lacking *STB*, showed high missegregation in the wild type and *cdc14-3* mutant strains (Type II + Type IV > Type I + Type III, S6A and S6B Fig). A characteristic feature of pSV5-*STB*, paucity of Type II + Type III cells (which signify contrary segregation between plasmid and *TEL V* or rDNA; Fig 4A and 4B), was not the case for pSV6-*ARS* (S6C and S6D Fig). In the wild type, Type II + Type III exceeded Type I + Type IV by ~20%, and the difference was opposite in the *cdc14* mutant. As non-disjunction tends to trap *TEL V* and rDNA in the mother nucleus, their segregation in the mutant approximates that of *ARS*-plasmids typified by their strong intrinsic mother bias [71]. Consequently, the shift from wild type to *cdc14-3* switches the distribution towards Type I + Type IV at the expense of Type II + Type III.

The pSV7-*CEN* plasmid segregated equally in most of the cells irrespective of the strain background (Type I + Type III >> Type II + Type IV; S7A and S7B Fig). As expected for normal *CEN* function, correlated segregation of pSV7-*CEN* with *TEL V* or rDNA was high in the wild type strain (Type I + Type IV >> Type II + Type III) (S7C and S7D Fig). Consistent with the adverse effect of Cdc14 inactivation on *TEL V* or rDNA segregation without disruption of general chromosome segregation, this strong correlation was absent in the *cdc14* mutant (Type I + Type IV < Type II + Type III) (S7C and S7D Fig).

To further verify the apparent distinction between an *STB*-plasmid and an *ARS*- or a *CEN*-plasmid in segregation under Cdc14 inactivation, we followed the single copy reporter plasmids (pSG1-*STB*, pSG1-*CEN* and pSG1-*ARS*) with respect to Nop1, a nucleolar histone glutamine methyl transferase, as an rDNA surrogate in the *cdc14* mutant strain (S8A and S8B Fig). Consistent with the results from Figs 4, S6 and S7, pSG1-*STB* alone showed the correlation with Nop1 in segregation (Type I + Type IV >> Type II + Type III) (S8C Fig; see also Fig 4). Segregation of pSG1-*CEN* was characterized by Type I +Type III (equal) >> Type II + Type IV (unequal), while the opposite was true for pSG1*-ARS* (Type II + Type IV > Type I + Type III) (S8D Fig).

Thus, Cdc14-assisted segregation of *TEL V* or rDNA also impacts plasmid segregation directed by the Rep1-Rep2-*STB* system. Neither *CEN*-mediated equal plasmid segregation (and by inference global chromosome segregation) nor high plasmid missegregation in the absence of a partitioning system is affected by the *cdc14* mutation. These distinctions are best appreciated by the sharp differences in the *p* values for pSG1-*STB* in Fig 4C and 4D from those for pSG1-*ARS* in S6C and S6D Fig and for pSG1-*CEN* in S7C and S7D Fig. The observed *STB*-plasmid behavior in the mutant stain is therefore not caused by some non-specific effect on cell physiology. The correlation between *STB*-plasmid missegregation and *TEL V* or rDNA missegregation is consistent with condensed chromosome loci being preferred sites for chromosome interaction by the 2-micron plasmid. Alternatively, the segregation of all three may share a condensin-dependent step triggered by Cdc14. The two possibilities are not mutually exclusive.

### Missegregation of Chr XII, but not Chr III, causes missegregation of the 2-micron plasmid

Faithful chromosome segregation in yeast, as in all eukaryotes, requires chromosome condensation, which facilitates compaction of chromosome arms, unlinking of catenated sister chromatids, and their organization into functional segregation units [72]. While condensation is easily visualized in the large chromosomes of plants and metazoans, more sensitive tools utilizing FRET or fluorescence quenching are needed to monitor condensation of the relatively short yeast chromosomes [64]. However, condensation of the rDNA locus on Chr XII (comprised of ~150 copies of a 9.1 kbp repeat unit; 1.5 Mbp DNA) has been revealed by standard microscopy in conjunction with FISH [73]. As already pointed out, segregation of the rDNA and *TEL* loci requires a Cdc14-dependent extra step of condensin recruitment and action. Interruption of this step perturbs the segregation of these loci and of the 2-micron plasmid, while chromosome segregation overall proceeds almost normally.

When gross missegregation of yeast chromosomes is induced by a conditional mutation, the majority of foci formed by a fluorescence-tagged multi-copy *STB*-plasmid segregates to the nuclear compartment containing the bulk of the chromosome mass [15, 31]. When the entire set of replicated sister chromatids is forced into either the mother or the daughter nucleus, a single-copy *STB*-plasmid is almost always localized in the chromosome-containing nucleus [32]. These observations are consistent with plasmid-chromosome interaction sites being distributed on more than one (perhaps all) chromosomes. However, the correlation between plasmid and Nop1 (rDNA) during segregation under Cdc14 inactivation suggests that missegregation of Chr XII, housing the rDNA array and critically dependent on condensin for segregation, is likely to affect 2-micron plasmid segregation more severely than missegregation of a smaller chromosome with less stringent dependence on condensin. We tested this hypothesis by following plasmid behavior in cells missegregating either Chr XII (one of the longest yeast chromosomes) or Chr III, which is roughly one-third as long.

**Fig 5:**
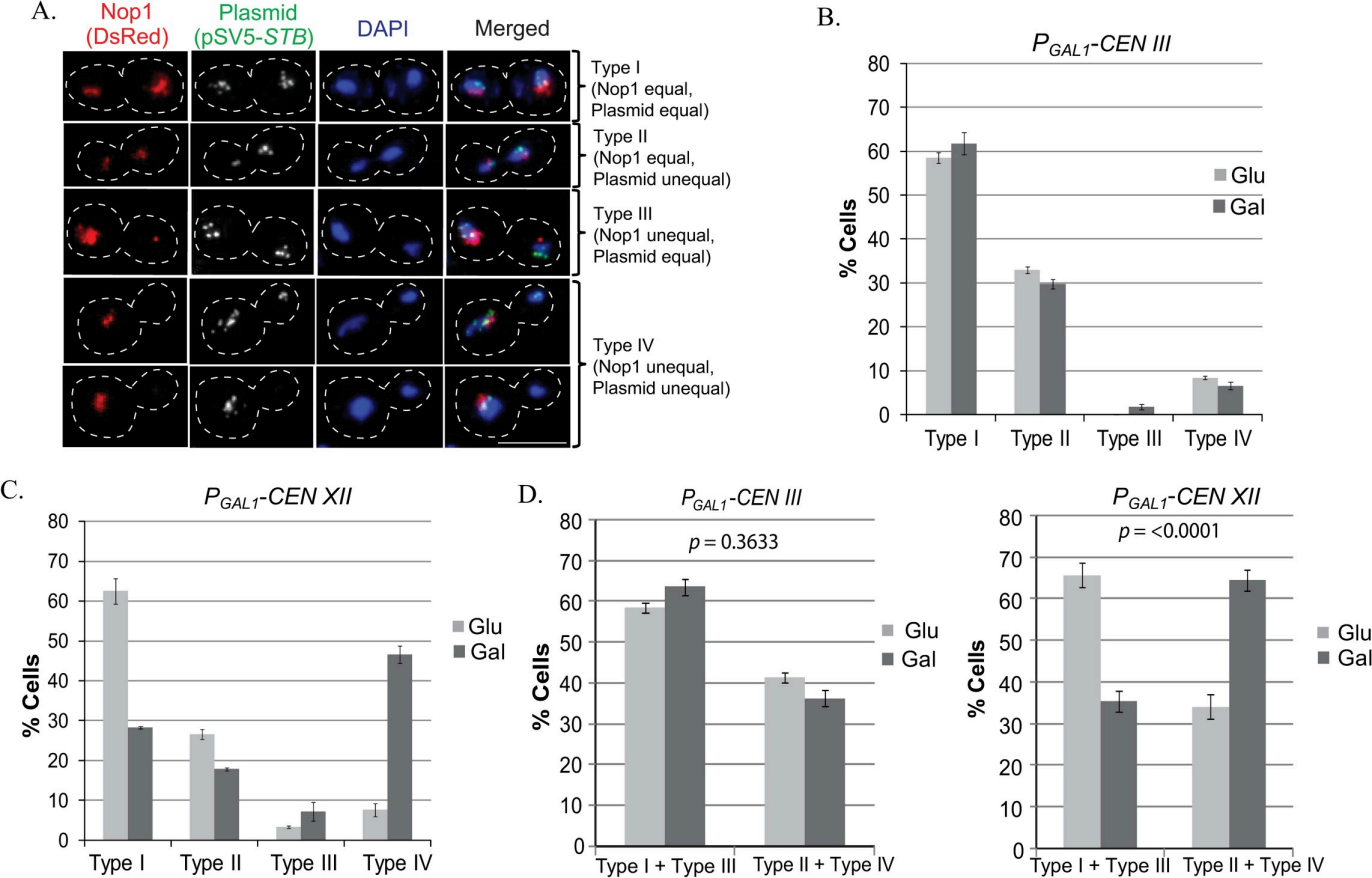
2-micron plasmid segregation in cells missegregating either Chr III or Chr XII. **A.** The experimental strains contained the *GAL1* promoter placed proximal to *CEN III* or *CEN XII* to drive high-level transcription through them under galactose induction. These strains were engineered to express GFP-LacI and Nop1 (DsRed) for fluorescence tagging pSV5-*STB* ([LacO]_256_) and Chr XII (rDNA), respectively. Plasmid and chromosome segregations were assayed in anaphase cells under uninduced (glucose; *CEN III* and *CEN XII* active) or induced (galactose; *CEN III or CEN XII* inactive) conditions. The four segregation patterns analyzed (Types I-IV) are illustrated by representative cell images. **B, C.** The cell fractions showing Type I-IV segregation are plotted for *CEN III* inactivation (**B**) and *CEN XII* inactivation (**C**). **D**. The data from (**B**) and (**C**) are replotted to highlight the differences in equal (Type I + Type III) and unequal (Type II + Type IV) plasmid segregation *vis a vis* Chr III or Chr XII segregation (normal or under *CEN* inactivation). At least 120 cells were analyzed for each set of assays depicted in (**B**) and (**C**). Statistical significance *p* value was estimated by Fisher’s exact test. Bar, 5 μm.

The pSV5-*STB* plasmid was introduced into isogenic strains in which either *CEN III* or *CEN XII* could be inactivated conditionally by *GAL* promoter driven transcription through them [74]. The extent of induced missegregation was nearly the same for Chr III and Chr XII (S9 Fig). The expression of GFP-LacI and Nop1 (DsRed) in these strains permitted plasmid segregation and Chr XII segregation (by Nop1 as proxy) to be monitored simultaneously against chromosome segregation as a whole visualized by DAPI staining. Missegregation of Chr XII, but not of Chr III, resulted in a significant increase in plasmid missegregation (Type II + Type IV > Type I + Type III) (Fig 5A–5D). Furthermore, in cells showing missegregation of both pSV5-*STB* and Nop1 (Type IV; Fig 5A), the majority of plasmid foci was present in the nucleus containing Nop1.

The linkage of the 2-micron plasmid with Chr XII but not Chr III is consistent with differences between these two chromosomes in the amount of condensin complex and/or the degree of chromatin condensation needed for their segregation. In the hitchhiking model, such differences may lead to preferred plasmid association with Chr XII. A potential plasmid target on Chr XII is the highly repeated rDNA locus, whose condensin utilization exceeds that of most other chromosome loci, except perhaps *TEL*s. The size advantage of chromosome XII over chromosome III should have only minimal effect on plasmid association/segregation, as it would be offset by the fifteen normally segregating chromosomes in each of the assays.

### Absence of condensin function disrupts faithful *STB*-plasmid segregation

The potential functional relationship between condensed chromatin and the 2-micron plasmid in segregation suggests two possible roles, neither one incompatible with the other, for the condensin complex in plasmid physiology. The multiple plasmid copies present within a chromosome-associated plasmid focus, which appears to be the unit in segregation [14, 15], may need to be condensed as a prerequisite for segregation. Or condensin may facilitate preferential plasmid association, and segregation in concert, with condensed chromosome loci. In support of the latter role, condensin is known to bridge DNA loci that are distal to each other on the genome. For example, it binds to tRNA genes dispersed throughout the chromosomes, and promotes their congregation into a cluster at the nucleolus [75]. In order to probe potential condensin requirement for 2-micron plasmid segregation, we compared the rates of missegregation of *STB*-plasmids with those of *CEN*- or *ARS*-plasmids following inactivation of the condensin component Brn1.

**Fig 6:**
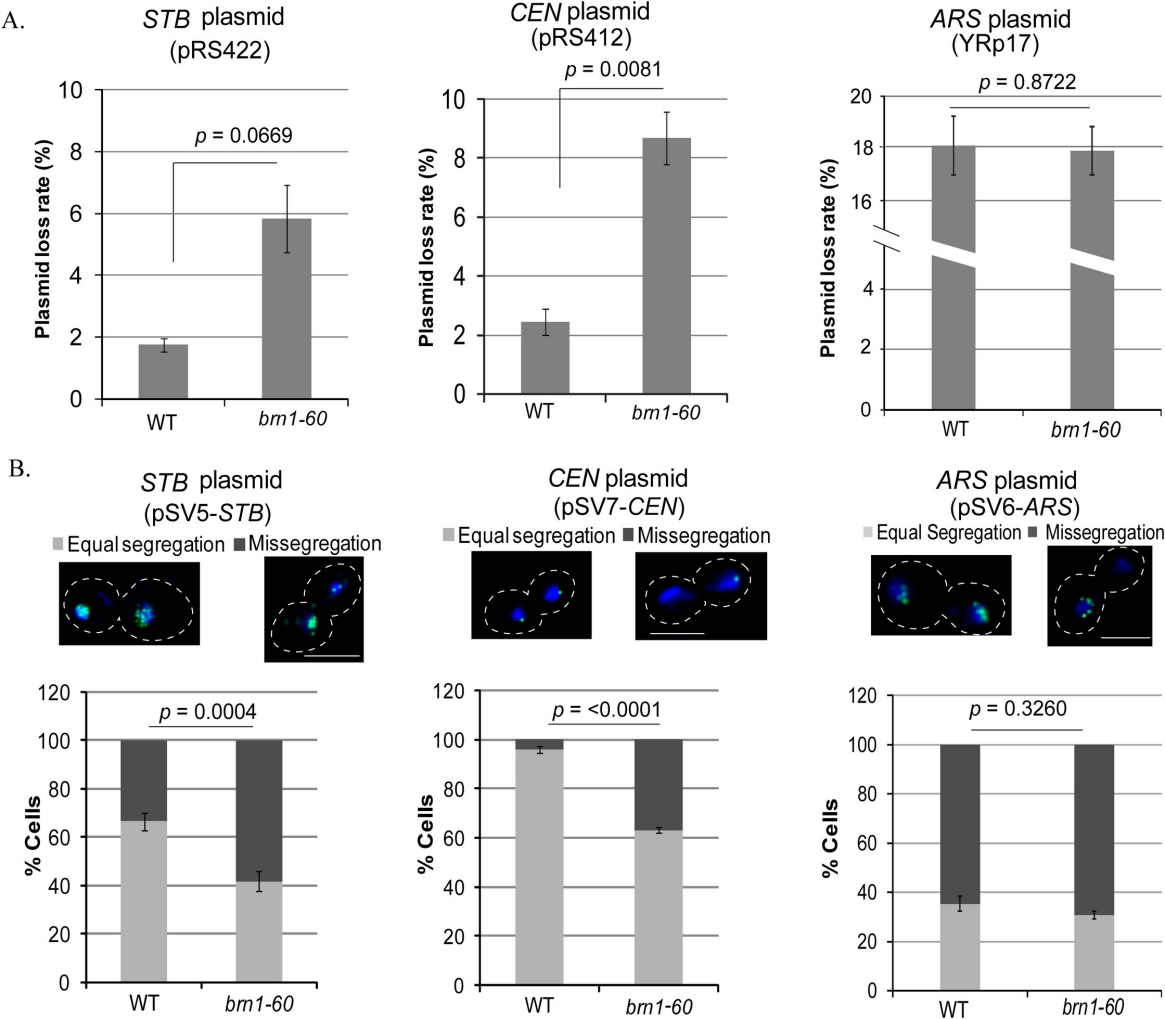
Missegregation of *STB*- and *CEN*-plasmids is increased in a condensin-defective strain relative to *ARS*-plasmids. **A.** Plasmid loss rates were estimated using three independent transformants each after 6–7 generations of non-selective growth at 36°C in the wild type and 4–5 generations in *brn1-60* strains (Materials and Methods). Approximately 200 colonies were scored for each transformant at the start and end points of non-selective growth. **B.** The segregation patterns of the fluorescence-tagged ([LacO]_256_-[GFP-LacI]) reporter plasmids were scored in the same strains as in (**A**). Approximately 100 cells were examined in each assay. Student’s t-test (two-tailed) (**A**) and Fisher’s exact test (**B**) were used to derive *p* values given in the plots. For a one-tailed t-test, based on the reasonable assumption that the *brn1* mutation is highly unlikely to improve plasmid stability from normal, the *p* values for the plots in **A** are: *STB*-plasmid (0.0334); *CEN*-plasmid (0.0040); *ARS*-plasmid (0.4361). Bar, 5 μm.

At 36°C, the loss of the *STB*-plasmid (pRS422; *ADE2*) and the *CEN*-plasmid (pRS412; *ADE2*) [76] was elevated in the temperature sensitive *brn1-60* mutant [77] over the wild type (Fig 6A). The *CEN*-plasmid instability is consistent with the previously demonstrated role of condensin in *CEN* function [62]. The inherently low stability of an *ARS*-plasmid (YRp17, *TRP1*) [78] was not further worsened in the mutant background (Fig 6A). These stability results were further verified by assaying the segregation of fluorescence-tagged *STB*-reporter and *CEN*-reporter plasmids (pSV5-*STB* and pSV7-*CEN*, respectively) in the wild type and mutant strains (Fig 6B). A fluorescence-tagged *ARS*-plasmid (pSV6-*ARS*), by contrast, was not altered in its segregation by the loss of condensin function (Fig 6B).

These results demonstrate the requirement of condensin during *STB*- and *CEN*-mediated plasmid segregation. However, they do not address whether condensin plays similar or distinct roles in the segregation functions of *STB* and *CEN*.

### Brn1 interacts with the 2-micron plasmid partitioning system

Since condensin is required for chromosome segregation, and the 2-micron plasmid is dependent on chromosomes for its own segregation, it is difficult to decipher whether condensin’s effect on the plasmid is direct or indirect. Proximity of the plasmid to condensed chromosome loci may lead to incidental plasmid-condensin association, and likely plasmid condensation as a result. Alternatively, condensin acquisition by the plasmid (and perhaps condensation) may be functionally related to the localization of a plasmid focus at condensed chromatin and its chromosome-associated segregation. One reasonable criterion for the direct involvement of condensin in 2-micron plasmid segregation would be its interaction with the Rep-*STB* system. Such interactions by host factors that assist plasmid partitioning have been demonstrated in previous studies through genetic interaction, chromatin immunoprecipitation (ChIP), and affinity enrichment-mass spectrometry assays [31, 34, 36, 38, 40]. We have now subjected condensin to similar tests using the Brn1 subunit of the complex as its representative.

**Fig 7:**
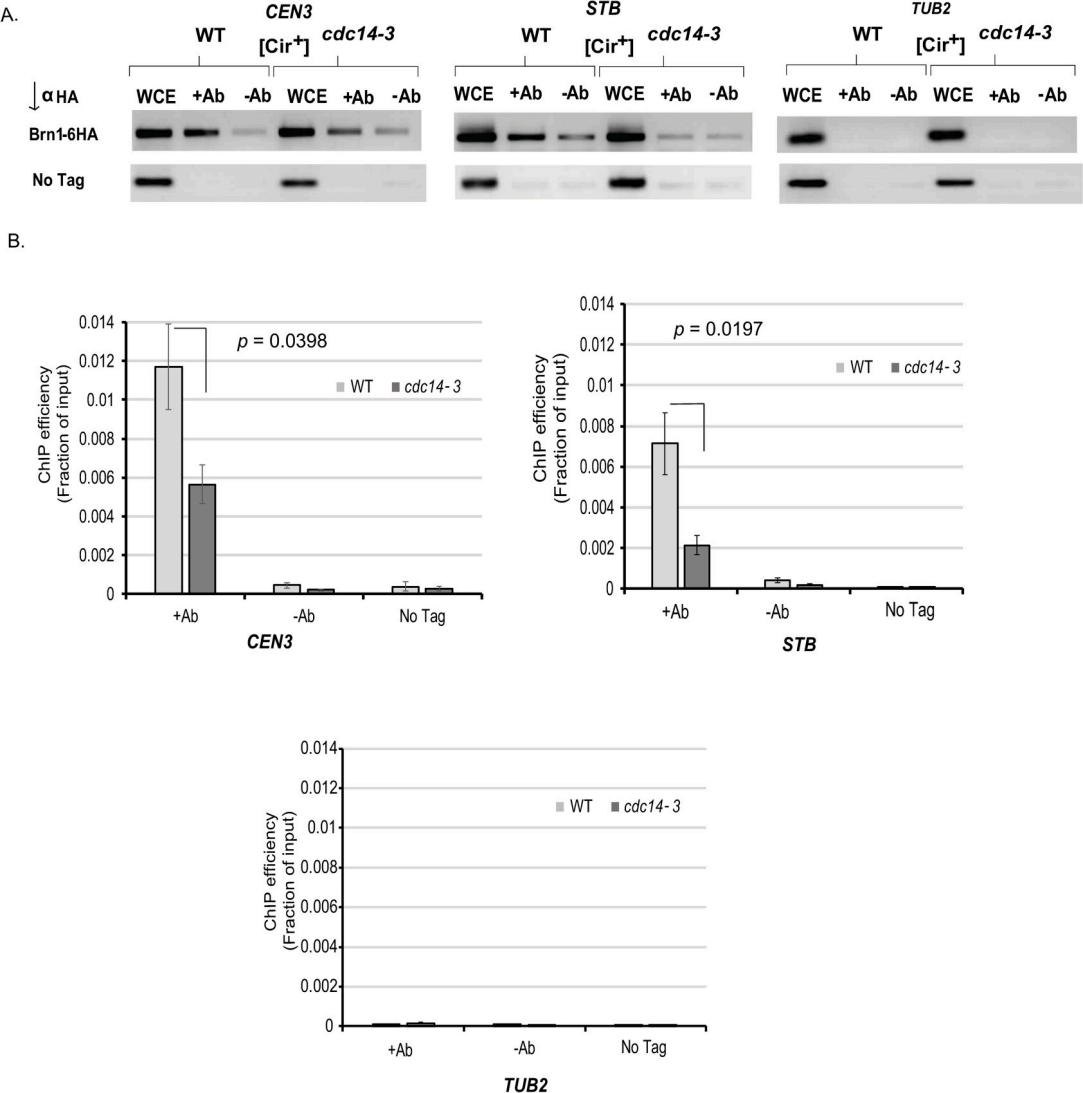
Brn1 associates with *STB*. **A.B.** ChIP analyses were performed using the indicated wild type and mutant strains ([Cir^+^]). *STB* (as a potential target for Brn1 association) was provided by the endogenous 2-micron plasmid in the [Cir^+^] strain. Brn1-6HA was immunoprecipitated using an antibody to the HA-tag. The ΔCT values between an experimental sample and the input DNA were derived from qPCR data after correcting for primer efficiency/amplification factor [109]. ChIP efficiency was quantitated as the fraction of the input DNA that was immunoprecipitated [ε^(-ΔCT)], where ε is the amplification factor). *CEN3* and *TUB2* loci were used as positive and negative controls for Brn1 binding, respectively. The gel images in (**A**) show ethidium bromide stained bands following fractionation of DNA amplified from immunoprecipitates by regular PCR. The graphs in (**B**) represent three independent experiments. The estimated *p* values are from two-tailed Student’s t-test.

**Fig 8:**
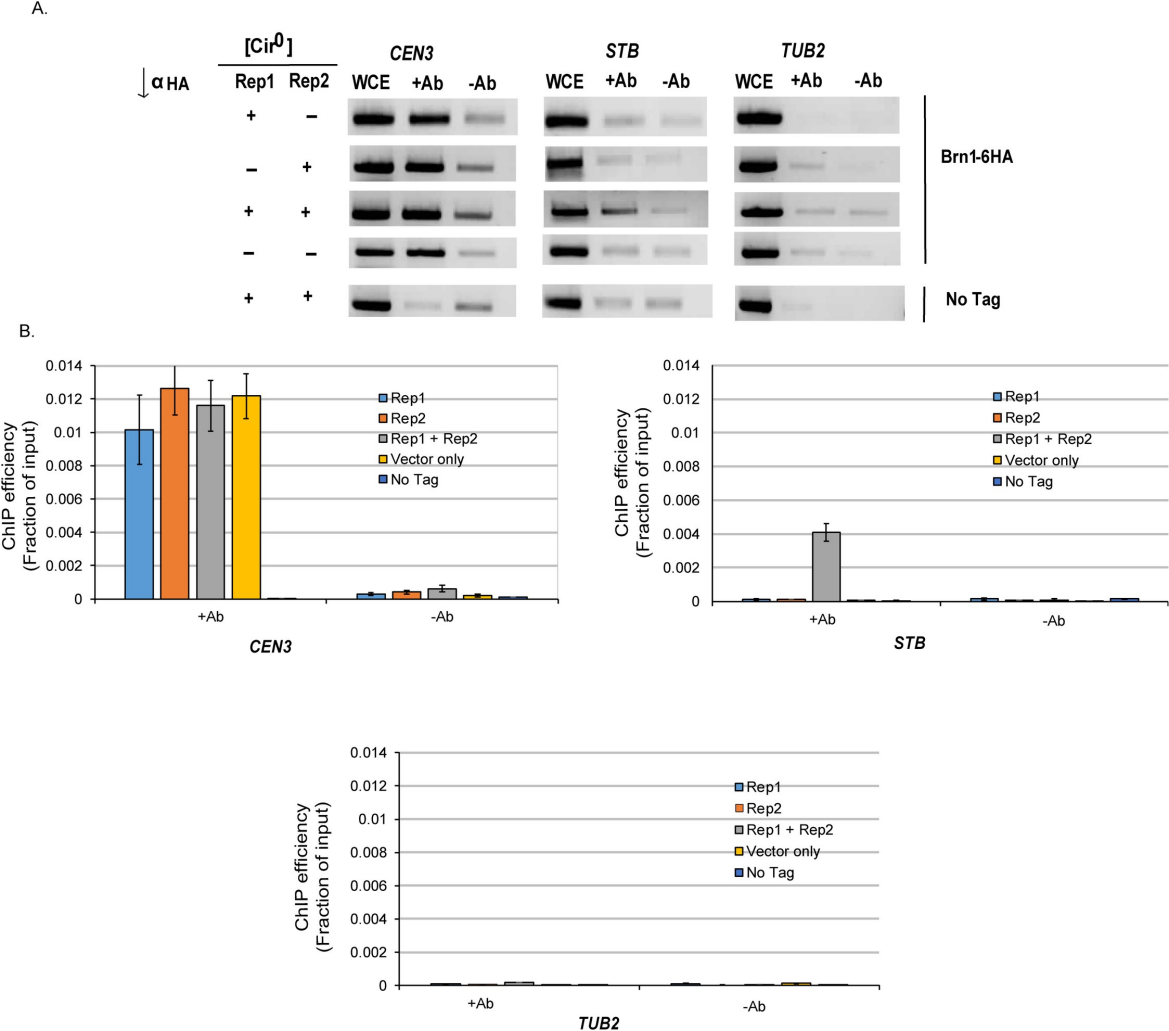
Brn1 association with *STB* is dependent on Rep1 and Rep2. **A**, **B**. ChIP analyses were performed as described under Fig 7 with the following differences. The experimental strain was [Cir^0^], and contained a single copy of *STB* integrated at the *ADE2* locus Derivatives of this strain contained integrations of *ADH1* promoter-controlled *REP1*, *REP2*, both *REP1* and *REP2* or neither (empty vector) at the *URA3* locus. Each assay set included three separate experiments.

Interaction between *STB* and Brn1 was ascertained by the enrichment of *STB* DNA during chromatin immunoprecipitation (ChIP) in a [Cir^+^] strain using anti-HA antibodies directed to Brn1-6HA (Fig 7A and 7B). The authenticity of Brn1-*STB* association was verified by the enrichment of *CEN III*, but not the tubulin locus *TUB2*, in the immunoprecipitate. *TUB2* has been designated as weak or negative in condensin localization [61]. Consistent with the higher missegregation of *STB*-plasmids under Cdc14 inactivation (Figs 4 and S8), ChIP in the *cdc14-3* mutant revealed a nearly complete absence of condensin at *STB* (Fig 7A and 7B). ChIP in a [Cir^0^] strain (lacking Rep1 and Rep2) failed to bring down a copy of *STB* integrated into the genome, with little or no effect on *CEN III* (Fig 8A and 8B). Expression of both Rep1 and Rep2 in this strain, but not Rep1 or Rep2 alone, restored Brn1-*STB* interaction (Fig 8A and 8B).

Rep1 and Rep2-mediated association of Brn1 with *STB* (Figs 7 and 8) suggests possible interaction of Brn1 with one or both of these partitioning proteins as implicated by unpublished observations mentioned in an earlier publication [15]. Results from two hybrid assays performed in a [Cir^+^] strain are consistent with this notion (S10 Fig). In principle, interaction with either Rep1 or Rep2 should result in Brn1 recruitment at *STB*, as Rep1 and Rep2 interact with each other [79–82] (S10 Fig). These two-hybrid results do not address whether Brn1 interaction with a Rep protein is dependent on the partner Rep protein or on host factor(s), including other component(s) of the condensin complex. Nevertheless, Brn1 interaction with at least one of the Rep proteins and the dependence of Brn1-*STB* association on both Rep proteins argue for condensin being an authentic host-coded 2-micron plasmid partitioning factor. The loss of Brn1 from *STB* upon Cdc14 inactivation and the rise in *STB*-plasmid instability upon Brn1 or Cdc14 inactivation bolster this possibility. The appropriation of the host’s DNA compaction and/or high-order DNA organization factors (condensin and cohesin, for example) by the 2-micron plasmid would be consistent with the evolutionary attunement of plasmid segregation to chromosome segregation.

## Discussion

We have characterized chromatin attributes exploited by the 2-micron plasmid for its localization and segregation during mitosis. Our findings suggest that condensed or condensin-enriched chromatin and/or silent heterochromatin-like regions of chromosomes are preferred targets for plasmid-chromosome association. Furthermore, the condensin complex interacts with the plasmid partitioning system, and facilitates equal plasmid segregation. A role for condensin/condensed chromatin in plasmid localization and segregation is consistent with the hitchhiking model, in which the two processes are intimately interconnected.

### 2-micron plasmid localization on yeast chromosomes

The chromosome-hitchhiking model for 2-micron plasmid segregation [13, 16, 32, 42] is based on a confluence of circumstantial, albeit persuasive, evidence. Strictly, plasmid association with a nuclear entity that segregates with the same characteristics as chromosomes cannot be ruled out. Direct demonstration of plasmid-chromosome association is impeded by the inability to resolve individual mitotic yeast chromosomes. Nevertheless, multi-copy and single-copy *STB*-reporter plasmids localize to yeast mitotic chromosome spreads in a Rep1-Rep2 dependent fashion, and colocalize with these proteins [31, 83]. In mammalian cells, co-expressed Rep1 and Rep2 form merged foci on mitotic chromosomes collectively, and along the arms of individual chromosomes in metaphase spreads [32]. Based on Rep1-Rep2 association with *STB* [15, 31, 80], and assuming the mammalian system to recapitulate the native yeast system, it follows that the 2-micron plasmid is tethered to yeast chromosomes with assistance from the Rep proteins. Meiotic yeast chromosome spreads, with considerably higher resolution than mitotic spreads, reveal *STB*-plasmid foci to be coalesced with a subset of Rep foci localized on chromosomes [42]. The preferential plasmid localization at *TEL*s or subtelomeric regions in these spreads differs from the reported *CEN* proximity of plasmid in mitotic cells [31]. Higher accuracy mapping has now localized the 2-micron plasmid near *CEN*s and *TEL*s in a majority of the cell population, with a clear bias towards *TEL*s (Figs 2 and S3). In over a third of the population, the plasmid location is distal to *CEN*s or a marked *TEL*. It is possible that these ‘other’ plasmid tethering sites on chromosomes (which may include *TEL*s that are not clustered with the marked one) share organizational features with *CEN* and *TEL*, for example, chromatin architecture and/or transcriptional quiescence.

### Functional link between *STB*-plasmid localization and segregation

Several observations suggest that Rep1-Rep2-assisted association of *STB*-plasmids precedes their chromosome-coupled segregation. Segregation of single-copy or multi-copy *STB*-plasmids is strongly correlated with that of chromosomes under gross chromosome missegregation induced by conditional mutations [15, 31], total entrapment of paired sister chromatids in mother or daughter nucleus by blocking cohesin disassembly [32], or under forced co-segregation of paired sister chromatids by inappropriate expression of the meiosis-specific monopolin complex during a mitotic cell cycle [16]. This correlated chromosome-plasmid segregation is lost in the absence of the Rep proteins (when a reporter plasmid behaves functionally as an *ARS*-plasmid). Equal plasmid segregation during meiosis is dependent on Rep1-Rep2-*STB* and on the bouquet protein Ndj1, which promotes plasmid association with telomeres [42].

The present study shows that the relationship between localization with respect to *CEN-TEL* (Figs 2 and 3) and segregation is quite different for *ARS*- and *STB*-plasmids. For *ARS*-plasmids, localization does not foster plasmid stability, which is quite low in wild type strains and remains low under treatments that alter chromosome segregation in specific ways (Figs 6 and S6). Blocking or delaying the disjunction of *TEL* DNA/rDNA by *cdc14* does not grossly disrupt *CEN* disjunction and hence bulk chromosome segregation. However, compromising *CEN* function/chromosome condensation by *brn1* impairs global chromosome segregation. In contrast to *ARS*-plasmids, *STB*-plasmids are sensitive to both *cdc14* and *brn1* (Figs 4 and 6). *STB*-plasmids also differ from *CEN*-plasmids whose segregation is affected by *brn1* but is relatively insensitive to *cdc14* (Figs 6 and S7).

The observed responses of *STB*-plasmids to distinct types of chromosome segregation defects, which set them apart from *ARS*-plasmids and also differentiate them from *CEN*-plasmids in certain respects, are consistent with their attachment to chromosome sites and such attachment being salient to plasmid segregation.

### A role for chromatin organization in the selection of plasmid tethering sites?

The 2-micron plasmid partitioning system may recognize a class of chromosome regions, including *TEL*s and *CEN*s, by their heterochromatin signature(s). The non-canonical heterochromatin of *S*. *cerevisiae* promoted by Sir proteins [84–87] induces transcriptional silencing at *TEL*s, rDNA and *HML*/*HMR* mating type loci, and enables *CEN*-mediated mitotic chromosome stability. Despite being genetically defined, the point *CEN*s of *S*. *cerevisiae* manifest a certain degree of epigenetic character, which is typical of regional *CEN*s. Established *CEN*s are functionally propagated through many generations in budding yeast strains carrying kinetochore mutations that block *de novo CEN* establishment [88]. The *TEL*- and *CEN*-proximal localization of the 2-micron plasmid is consistent with a possible origin of *CEN* from *TEL* [89]. They do share organizational or functional features in certain biological contexts. In the fission yeast, *CEN*-centrosome contact can replace *TEL*-centrosome contact for successful meiosis [90]. The plasmid presence at *CEN*-*TEL* may be promoted by epigenetic mark(s) conserved during their evolutionary trajectories, which might also be retained within *CEN*-adjacent *CEN*-like regions (*CLR*s) [48].

### 2-micron plasmid missegregation upon Cdc14 inactivation

The functional relevance of *TEL* proximity of the 2-micron plasmid on chromosomes is upheld by the increase in plasmid missegregation when *TEL* (and rDNA) disjunction is impeded by inactivating Cdc14. However, total missegregation of all plasmid foci in tandem with *TEL* or rDNA (represented by Nop1) is rarely seen (Fig 4). This is the expected outcome if plasmid tethering sites were present at chromosome locales other than *TEL*s, including *CEN*-proximal regions, as revealed in the localization assays. According to a prior study, when the entire set of chromosomes is confined to either the mother or daughter nucleus, there is near perfect correlation between an *STB*-plasmid and chromosomes in their localization [32]. Taken together, these results support a non-random distribution of plasmid tethering sites on chromosomes, *TEL*s or nearby loci being high density or high affinity sites. Cdc14 inactivation has pleiotropic consequences, one of which is compromised spindle integrity [91, 92]. The mitotic spindle and spindle-associated proteins play a role, even if indirect, in 2-micron plasmid segregation [31, 34, 35]. Hence, potential contribution of spindle defect to plasmid missegregation under loss of Cdc14 function cannot be ruled out. However, such an effect is likely modest, as overall chromosome segregation (revealed by DAPI staining) appears to be normal in our assays. Furthermore, *STB* plasmid missegregation can also be induced by inactivating the condensin component Brn1, which is not known to be involved in spindle function.

### A role for the condensin complex in 2-micron plasmid segregation

As noted, Cdc14-promoted condensin recruitment late in the cell cycle at rDNA and *TEL*s is a pre-requisite for their proper segregation [68, 70]. The possibility that this instalment of condensin resolves rDNA sisters independently of chromosome condensation and Topo II activity has been raised [70]. However, full condensation of rDNA, approximately equal to that of condensed euchromatin, is completed only in anaphase [68]. Resolution of the interlinks between rDNA sisters by recruitment of Topo II in a condensin dependent step, or remodeling of the interlinks by condensin to a configuration favored by Topo II, has not been ruled out [68, 93]. The increased missegregation of the 2-micron plasmid in a condensin mutant (Fig 6) may result from a direct effect of condensin on the plasmid itself, or an indirect effect manifested through chromosomes on which it hitches a ride.

By being a chromosome appendage, the plasmid becomes subjected to the same physical and conformational constraints that a chromosome experiences during segregation. Chromosome condensation and plasmid condensation may thus be coincidental events. However, the interaction of Brn1 with the Rep proteins and its localization at *STB* with Rep protein assistance (Figs 7,8 and S10) would be consistent with a more direct role for condensin in plasmid segregation. Interestingly, Brn1 association with *STB* is reduced by Cdc14 inactivation (Fig 7). Perhaps Cdc14 dependent condensin assembly is shared by repeated loci that include rDNA and *TEL*s on chromosomes and the extra-chromosomal (but chromosome-associated) 2-micron plasmid. Cohesin-mediated pairing of plasmid sisters [31, 39], in conjunction with possible condensin-assisted DNA compaction, may promote the organization of replicated plasmid molecules into two equally populated sister clusters that tether symmetrically to sister chromatids. Such a mechanism is consistent with the segregation pattern observed for single-copy *STB* plasmids whose sister copies segregate one-to-one in most cells [12, 17, 32]. Condensin associated with *STB* may also serve to recruit Topo II to the plasmid and/or assist decatenation of plasmid sisters. Topo II inactivation causes an accumulation of replicated 2-micron plasmids as catenanes [94].

### Chromosome association by selfish DNA elements

The high fidelity of chromosome segregation is likely the driving force behind the convergence by the yeast plasmid and viral episomes, whose respective hosts diverged ~1.5 billion years ago, on exploiting chromosome segregation for self-preservation [3, 14, 95–97]. The *TEL*- and *CEN*-proximal plasmid localization in mitotic cells suggests a propensity for plasmid tethering sites to be sparsely populated by genes or organized into silent chromatin. It is noteworthy that the portion of *STB* proximal to the 2-micron plasmid replication origin, and critical to plasmid partitioning, resides in a transcription-free zone [98]. The origin-distal portion of *STB* contains a ‘silencer box’, and is important in protecting *STB*-distal from readthrough of neighboring transcription. Furthermore, *STB* can functionally replace the chromosomal silencer sequences adjacent to the *HML* yeast mating type locus [99]. Strikingly, the viral tethering sites on mammalian chromosomes also appear to be enriched for condensed/repressive chromatin that include pericentromeric, peritelomeric and rDNA loci [95, 100–106]. Such localization may be advantageous to a selfish DNA element by lowering the prospect of its dislodgement from chromosomes due to gene activity and by protecting the host against perturbations of gene functions.

The present studies, however, do not rule out other chromatin features that might be utilized by the 2-micron plasmid for chromosome tethering. A potential additional plasmid strategy for targeting more readily accessible chromatin may account for *TEL*/*CEN*-distal plasmid localization. Maximizing its options to remain chromosome-associated would be beneficial to the plasmid, and to any extra-chromosomal element, that propagates itself by chromosome hitchhiking.

## Materials and methods

### Yeast strains and plasmids

The yeast strains used in this study (W303 background) and their relevant genotypes are listed in S1 Table. Strains containing or lacking the native 2-micron plasmid are referred to as [Cir^+^] or [Cir^0^], respectively. The [Cir^+^] designation does not include [Cir^0^] strains transformed with 2-micron derived or other artificial plasmid constructs. In the strains for chromatin immunoprecipitation (ChIP) assays, the *BRN1* locus on Chr II was tagged with 6HA by inserting a PCR amplified DNA fragment containing the epitope tag via homologous recombination [107]. Gene fusion and expression of the tagged proteins were verified by PCR using total extracted DNA as template, and by immunostaining chromatin spreads with HA-antibody, respectively.

The plasmid constructs employed in this work and their salient features are summarized in S1 Table. The plasmid pRS402CFP-LacI (*ADE2*) was obtained by replacing the coding region for GFP in pRS402GFP-LacI [15] with that of CFP.

### Tagging *TEL VII* with TetO

CRISPR (Cas9-sgRNA) technology was used to tag *TEL VII* with a [TetO]_n_ array in a strain expressing [TetR-GFP] and containing [TetO]_448_ inserted at *TEL V* [27]. The PCR amplified DNA containing [TetO]_n_ (obtained from pRS306-[TetO]_224_ as template) flanked by chromosome homology at either end was inserted between *IMA1* and *MAL13* genes on the right arm of Chr VII (~22 kbp from the end). Viable colonies obtained following transformation were screened by fluorescence microscopy to identify successful integrants. Based on the sizes of PCR products amplified using isolated total DNA, the number of [TetO] copies was ≤ 50, and varied in individual transformants. One transformant showing two green fluorescent foci (bright *TEL V* and faint *TEL VII*) in most individual cells was saved, and used for further analyses.

### Cell cycle synchronization

Cells were arrested in G1/S with α-factor, and released into the cell cycle by washing off the pheromone. Reporter plasmids were localized in the arrested cells and following their release. Cells harvested at intervals during cell cycle progression were examined by microscopy. Metaphase and anaphase cells were identified by their characteristic morphological features, as described previously [17, 40].

### Plasmid stability assays

Cultures of transformant colonies containing a reporter plasmid were grown overnight in selective liquid medium, and were diluted into YPDA medium (non-selective). The inocula were grown for ‘n’ generations in non-selective media (n varied from 5 to 10, depending on the experiment). The fractions of plasmid containing cells in the population at the start (f_0_) and at the end (f_n_) of growth in the non-selective media were estimated by plating equal aliquots of the cultures on selective and non-selective plates. The plasmid loss rate per generation was estimated as ‘i’ = (1/n) x ln(f_0_/f_n_) [98].

### Plasmid segregation under induced missegregation of chromosomes

For inducing Chr III or Chr XII to missegregate in galactose medium, the *GAL1* promoter was inserted immediately upstream of *CEN III* or *CEN XII* as described previously [74]. Cells harboring the *GAL-CEN* cassette were grown till mid-log phase in SC (-Leu, -Trp, raffinose) medium at 30°C before being transferred to SC (-Leu,-Trp) medium supplemented with either 2% galactose or 2% glucose for 5 hr at 30°C. Cells were harvested, stained with DAPI, and reporter plasmids ([LacO]-[GFP-LacI]) and rDNA (DsRed-Nop1) were observed by fluorescence microscopy to follow the segregation pattern of plasmid with respect to rDNA. In strains lacking a reporter plasmid, segregation of Chr III was assayed by fluorescence-tagging at the *LEU2* locus using [LacO]-[GFP-LacI]; that of Chr XII was followed by tracking rDNA marked with DsRed-Nop1.

### Di-hybrid assay

The strain for di-hybrid assays contained the *HIS3* reporter gene regulated by the *GAL1* UAS (upstream activation sequence) [108]. The bait and prey proteins to be tested were fused to the Gal4 DNA binding and Gal4 activation domains, respectively, and their expression was controlled by the *ADH1* promoter. Positive bait-prey interaction resulting in enhanced *HIS3* transcription was revealed by colony growth on medium lacking histidine and supplemented with 0.25 mM 3-AT (3-amino-1,2,4-triazole) to inhibit His3 activity resulting from basal expression. A positive response by a pair of test proteins was verified by reciprocally swapping the DNA binding and activation domains between them. Expression of the binding or activation domain alone (without fusion to the bait or prey) provided the negative controls. Interaction between Rep1 and Rep2 was used as positive control.

### Chromatin Immunoprecipitation assays

Chromatin Immunoprecipitation (ChIP) assays were performed as described previously [109, 110]. The *STB* from the native 2-micron circle provided the potential target for Brn1 association in the [Cir^+^] strain. In the [Cir^0^] strain used for ChIP, a copy of *STB* was integrated into the genome at the *ADE2* locus. Derivatives of this strain were constructed to express either Rep1, Rep2 or Rep1 and Rep2. Expression was driven by the *ADH1* promoter from cassettes integrated at the *TRP1* locus (for *REP1*) and *URA3* locus (for *REP2* or *REP1* and *REP2*). Brn1 fused to 6HA at the carboxyl-terminus was immunoprecipitated using a polyclonal antibody to HA (Rabbit polyclonal HA11, Abcam, UK). The specificity of the association was verified using a chromosomal locus ‘*TUB2*’ designated to be weak or negative in condensin association [61]. Brn1 with no tag and mock immunoprecipitation (no antibody) provided additional negative controls. The relative amounts of immunoprecipitated DNA were quantitated using qPCR [110]. Corrections were applied to account for primer efficiencies below 100% (amplification factors < 2.0) using standardization graphs of CT values against dilutions of the input DNA. The amplification factor ε was estimated as 10^(-1/slope) of the regression line, and the primer efficiency E (%) as {[10^(-1/slope)]– 1} x 100. The fraction of immunoprecipitated DNA in a ChIP sample relative to the input DNA was calculated as ε^∧^(−ΔCT), ΔCT = CT (ChIP)–[CT (Input)–log_ε_(Input dilution factor)]. The primers used for PCR amplification are listed in S2 Table under ‘Supporting Information’.

### Fluorescence microscopy and distance measurement

Fluorescence microscopy was performed in live or mildly fixed cells. Fixing was performed in *p*-formaldehyde (4% v/v) by incubating on ice for 5–10 min. After two washes with 0.1 M phosphate buffer (pH 7.4), cells were imaged using a Zeiss Axio Observer Z1 fluorescence microscope and the Axiovision software. Images were acquired using the z-stack mode, with a 0.20 μm interval between two successive planes [111]. They were opened in Imaris software (Bitplane, Imaris 8.0.2), and ‘slice tool’ was utilized to measure the spacing between the centroids of two fluorescent foci [112]. The distances were grouped into two types, ≤ 0.5 μm and > 0.5 μm.

### Segregation of fluorescence-tagged multi-copy reporters

The method for estimating segregation of multi-copy fluorescence-tagged reporter plasmids (Figs 4, 5 and S6–S8) was similar to that described previously [15]. Plasmid foci in the two nuclear compartments of an anaphase cell were counted by scanning stacks along the Z-axis, and were categorized as ‘equal’ or ‘unequal’. These estimates are subject to some intrinsic error from the potential overlap of foci and variations in plasmid copy number among foci.

### Biological replicates and statistical methods

The quantitative data shown in Figures and Supplementary Figures were obtained from two to three biological replicates. The N values given in histogram plots are the total number of cells analyzed from combined replicates of individual assays. Data analyzed using GraphPad Prism software (8.4.3) are presented as mean ± SE. Statistical significance was estimated using Student’s T-test, Chi-square test or Fisher’s exact test, as appropriate.

## Supporting information

S1 Fig The functional states of *CEN* and *STB* in plasmid pSG1 under distinct experimental contexts. In the pSG1 plasmid, schematically diagrammed at the top, the centromere (*CEN*) is placed immediately downstream of the *GAL* promoter. The segregation status of the plasmid in a [Cir^0^] or [Cir^+^] host strain under glucose or galactose as the carbon source is tabulated below. *CEN* is active in both hosts when the promoter is turned off by glucose repression. It is inactivated by galactose-induced transcription. The Rep1 and Rep2 proteins provided by the native 2-micron circle of the [Cir^+^] strain keep *STB* active, regardless of the carbon source. In the [Cir^0^] strain, lacking Rep1 and Rep2, *STB* is inactive. In the [Cir^0^]/galactose context, neither *CEN* nor *STB* is active. As a result, pSG1 segregates as an *ARS* plasmid. The active and inactive states of *CEN* or *STB* are indicated by ‘+’ and ‘-‘, respectively.
(TIF)

S2 Fig The localization patterns of SPB, *CEN V* and *TEL V* during different stages of the mitotic cell cycle. In the experimental strain, SPB was marked with red fluorescence using Spc42 fused to m-cherry. *CEN V* and *TEL V* were tagged by green fluorescence via [TetO]_n_-[TetR-GFP] interaction. These two loci were distinguished by their differential brightness ([TetO]_224_ at *CEN* and [TetO]_448_ at *TEL*). **A.** The representative images from fixed cells (left) and their schematic illustrations (right) denote the positions of the three nuclear landmarks at different stages of the cell cycle. The arrows and arrowheads point to *CEN V* and *TELV*, respectively. **B.** The histograms show distance measurements of *CEN V* and *TEL V* from SPBs divided into two categories (≤ 0.5 μm and > 0.5 μm). Statistical significance *p* value was estimated using the Student’s t-test (two-tailed). Bar, 5 μm.
(TIF)

S3 Fig Preferential localization of pSG1-*STB* near *TEL*s revealed at the anaphase stage of mitosis. **A**. The representative images (rows at the left) depict a subset of early anaphase cells ([Cir^+^]; galactose grown) in which pSG1-*STB* was mapped with respect to well resolved *CEN V* (indicated by the arrows) but unresolved or closely spaced *TEL V* foci. Fluorescence tags: pSG1-*STB*, [LacO]_256_-[CFP-LacI]; *CEN V*, [TetO]_224_-[TetR-GFP]; *TEL V*, [TetO]_448_-[TetR-GFP]; SPB, (Spc42-mCherry). The pSG1-*STB* positions were classified into three types as idealized by the schematic diagrams at the right. **B**. The plot shows the relative frequencies of the three types. Placement in the ≤ 0.5 μm class required both plasmid copies to satisfy this criterion. The > 0.5 μm class included cells in which one or both of the plasmid copies exceeded the cut-off distance. Bar, 5 μm.
(TIF)

S4 Fig Relative distances of pSG1-*STB*, pSG1-*ARS* and pSG1-*CEN* from *CEN V* and *TEL V* within the 3D nuclear space. **A**. The positioning of the centroids of the fluorescent foci by Z-series sectioning of the nucleus is schematically shown. Images were captured from the [Cir^0^] experimental strain containing fluorescence-tagged SPB, *CEN V* and *TEL V* grown in glucose (pSG1-*CEN*) or galactose (pSG1-*ARS*) or the isogenic [Cir^+^] strain grown in galactose (pSG1-*STB*). Image analysis was performed using Imaris ‘Slice’ tool (see Materials and Methods for details). **B**-**D**. Plasmid distances from *CEN V* and *TEL V* are shown as dot plots. The data are based on scoring of at least 74 cells for each plasmid.
(TIF)

S5 Fig Simultaneous localization of *pSG1-STB* with respect to *TEL V* and *TEL VII*. The analysis was similar to that described in the legend to Fig 2. The pSG1-*STB* plasmid ([Cir^+^]; galactose; [LacO]_256_-[CFP-LacI]) was localized with respect to *TEL V* and *TEL VII* in fixed G1/S cells. The *TEL V* and *TEL VII* tagged by [TetO_n_]-[TetR-GFP] were distinguished by the difference in the brightness of their fluorescence (*TEL V* >> *TEL VII*). **A**. The location of pSG1-*STB* was scored in the subset of cells in which *TEL V* and *TEL VII* were clearly resolved (>0.5 μm). **B**. The spacing of *TEL V* and *TEL VII* from each other was estimated.
(TIF)

S6 Fig *ARS*-plasmid segregation when missegregation of *TEL*s or rDNA is induced by Cdc14 inactivation. **A**, **B**. The experimental protocols were similar to those described under Fig 4, except that the reporter plasmid employed was pSV6-*ARS*. Plasmid segregation was assayed in conjunction with that of *TEL V* (**A**) or of rDNA (**B**). Fluorescence tags: pSV6-*ARS*, [LacO]_256_-[CFP-LacI]; *TEL V*, [TetO]_448_-[TetR-GFP]; rDNA, [TetO]_448_-[TetR-GFP]. The images of cell types (I-IV) and histogram plots of their quantitative analysis are arranged similarly to those in Fig 4. The (Type I + Type IV) and (Type II + Type III) plots represent plasmid segregation correlated and uncorrelated, respectively, with *TEL V* (**C**) and rDNA (**D**) segregation. The data for each assay set in wild type and *cdc14-3* cells were derived from a minimum of 148 cells. Statistical analysis employed Fisher’s exact test. Bar, 5 μm.
(TIF)

S7 Fig *CEN*-plasmid segregation under Cdc14 inactivation to induce missegregation of *TEL*s and rDNA. **A**, **B**. The assays in the wild type and *cdc-14-3* strains (otherwise isogenic) were carried out as described in the legend to Fig 4 (also S6 Fig) with pSV7-*CEN* as the reporter plasmid. Fluorescence tags: pSV7-*CEN*, [LacO]_256_-[CFP-LacI]; *TEL V*, [TetO]_448_-[TetR-GFP]; rDNA, [TetO]_448_-[TetR-GFP]. The Types I-IV segregation represented by the cell images shown at the top are quantitated in the histogram plots below them. The (Type I + Type IV) and (Type II + Type III) plots represent plasmid segregation correlated and uncorrelated, respectively, with *TEL V* (**C**) and rDNA (**D**) segregation. At least 150 cells were scored in each set of assays for the wild type and *cdc14-3* cells. Fisher’s exact test was used to estimate statistical significance. Bar, 5 μm.
(TIF)

S8 Fig Segregation of single copy reporter plasmids (pSG1-*STB*, pSG1-*CEN*, and pSG1-*ARS*) with respect to Nop1 under Cdc14 inactivation. The partitioning status of the pSG1-reporter plasmid was manipulated as indicated in S1 Fig to obtain pSG1-*STB*, pSG1-*CEN* and pSG1-*ARS*. Segregation assays were performed at the non-permissive temperature (33°C). Fluorescence tags: pSG1, [LacO]_256_-[CFP-LacI]; Nop1 (DsRed). Segregation types I-IV represented in the cell images at the left (**A**) are quantitated in histogram plots at the right (**B**). The data shown in (**C**) and (**D**) are reformatted from those in (**B**). The plots in (**C**) highlight the distinctions between pSG1-*STB* and pSG1-*CEN* or pSG1-*ARS* in Nop1-correlated segregation (Type I + Type IV) and Nop1-uncorrelated segregation (Type II + Type III). In (**D**), overall plasmid segregation, Nop1-correlated or not, is displayed equal (Type I + Type III) or unequal (Type II + Type IV). For each plasmid type, over 200 cells were analyzed. Pairwise comparisons of pSG1-*STB* with pSG1-*ARS* or pSG1-*CEN* (**C**) by Fisher’s exact test showed statistical significance in each case (*p* < 0.0001). This was not the case for the pSG1-*ARS* and pSG1-*CEN* pair, *p* = 0.392. The Type I-Type IV distributions were statistically indistinguishable for the pSG1 plasmids (this figure) and the corresponding pSV set of plasmids (Figs 4, S6 and S7) under Cdc14 inactivation. For each pairwise comparison, chi-square test gave *p* > 0.05: pSV5-*STB* and pSG1-*STB* (*p* = 0.1350); pSV6-*ARS* and pSG1-*ARS* (*p* = 0.1618) and pSV7-*CEN* and pSG1-*CEN* (*p* = 0.0814). Bar, 5 μm.
(TIF)

S9 Fig Segregation of Chr III and Chr XII under Glucose (Glu) and Galactose (Gal) conditions. **A**. In the experimental strains, lacking a fluorescence-tagged reporter plasmid, the *GAL1* promoter-controlled *CEN III* or *CEN XII* was kept active or inactive in glucose or galactose, respectively. The strain with the conditionally active *CEN III* was engineered to express GFP-LacI, and contained a [LacO]_256_ insertion within Chr III. The strain with the conditionally active *CEN XII* was engineered to express fluorescence-tagged Nop1 (DsRed). The images show equal segregation and missegregation of Chr III and Chr XII, as followed by green and red fluorescence, respectively. **B**. The data from the analysis of at least 120 cells for each assay are plotted. Bar, 5 μm.
(TIF)

S10 Fig Brn1 interacts with Rep1 and Rep2 in dihybrid assays in [Cir^+^] strains. The dihybrid system based on the *HIS3* reporter [108] is schematically diagrammed at the top (AD, activation domain; BD, binding domain). The expression vectors for *ADH1* promoter-controlled AD- and BD-fusions were maintained by selection for the *LEU2* and *TRP1* markers, respectively, that they harbored. The indicated protein-protein interactions were assayed on SC-Leu-Trp-His and SC-Leu-Trp-His plates with or without added 3-AT. The interaction between Rep1 and Rep2 served as the positive control (last two rows of the top panel). The empty AD and BD vectors are indicated by ‘-‘.
(TIF)

S1 Table Strains and plasmids. The list of yeast strains and plasmids used in this study.
(DOCX)

S2 Table The list of primer sequences. The DNA sequences of the primers used in Figs 7 and 8.
(DOCX)
