# Supplementary figures and images for: The selfish yeast plasmid utilizes the condensin complex and condensed chromatin for faithful partitioning
Source: PLoS Genet. 2021 Jul 16;17(7):e1009660. doi: 10.1371/journal.pgen.1009660 (PMC8318298; doi:10.1371/journal.pgen.1009660)

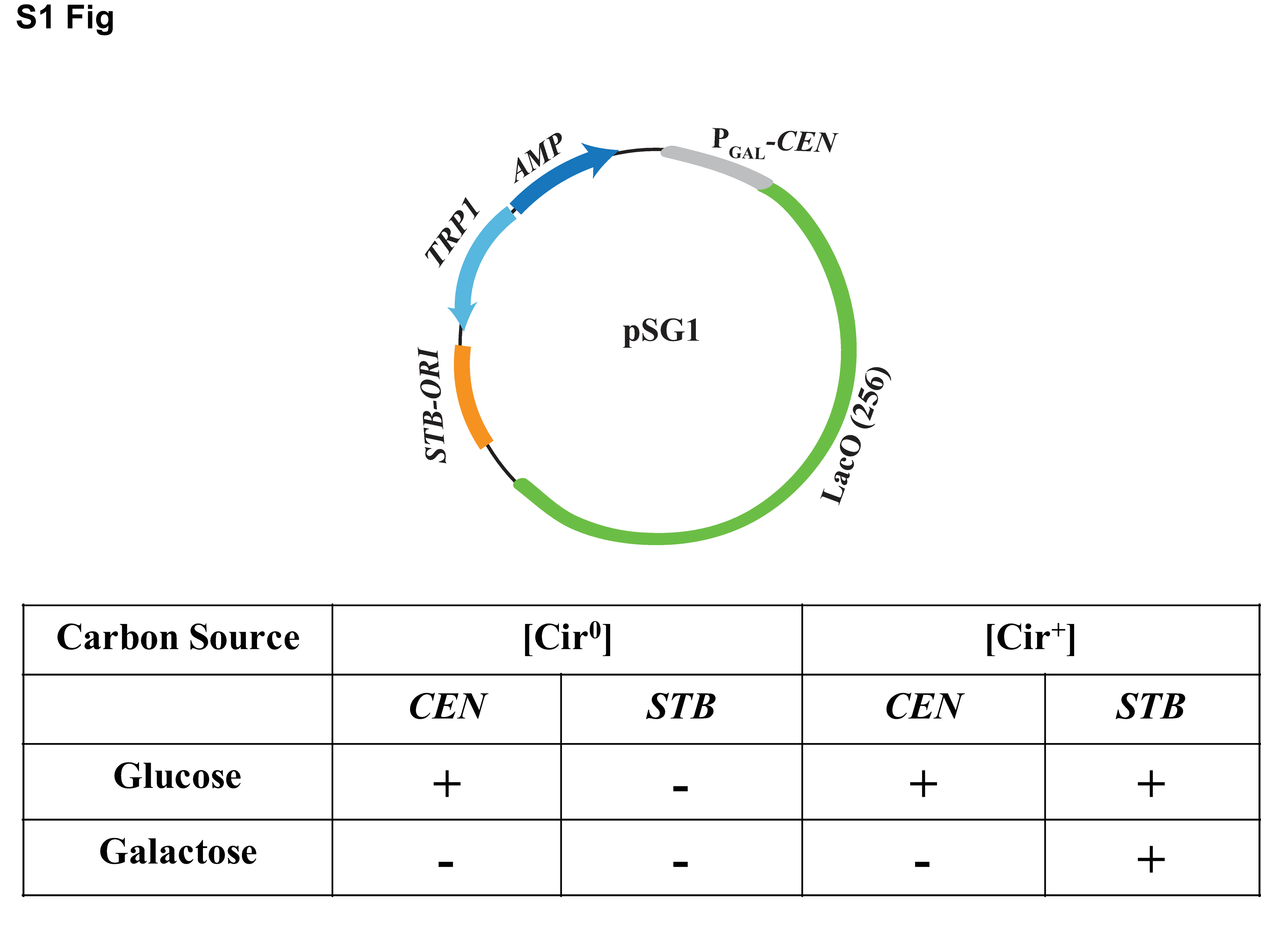

Supplement: S1 Fig — In the pSG1 plasmid, schematically diagrammed at the top, the centromere (CEN) is placed immediately downstream of the GAL promoter. The segregation status of the plasmid in a [Cir0] or [Cir+] host strain under glucose or galactose as the carbon source is tabulated below. CEN is active in both hosts when the promoter is turned off by glucose repression. It is inactivated by galactose-induced transcription. The Rep1 and Rep2 proteins provided by the native 2-micron circle of the [Cir+] strain keep STB active, regardless of the carbon source. In the [Cir0] strain, lacking Rep1 and Rep2, STB is inactive. In the [Cir0]/galactose context, neither CEN nor STB is active. As a result, pSG1 segregates as an ARS plasmid. The active and inactive states of CEN or STB are indicated by ‘+’ and ‘-‘, respectively. (TIF) [file pgen.1009660.s001.tif]

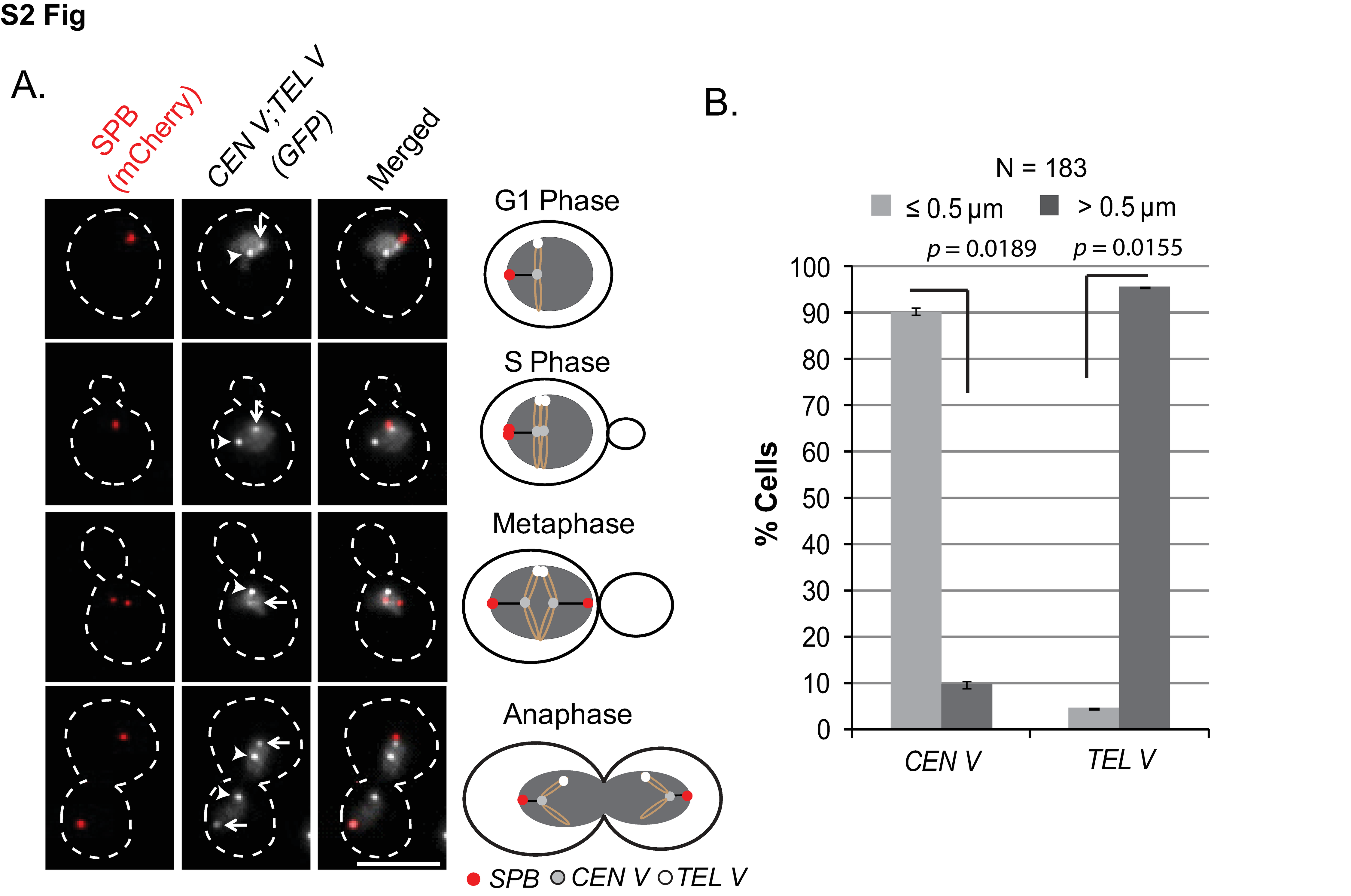

Supplement: S2 Fig — In the experimental strain, SPB was marked with red fluorescence using Spc42 fused to m-cherry. CEN V and TEL V were tagged by green fluorescence via [TetO]n-[TetR-GFP] interaction. These two loci were distinguished by their differential brightness ([TetO]224 at CEN and [TetO]448 at TEL). A. The representative images from fixed cells (left) and their schematic illustrations (right) denote the positions of the three nuclear landmarks at different stages of the cell cycle. The arrows and arrowheads point to CEN V and TELV, respectively. B. The histograms show distance measurements of CEN V and TEL V from SPBs divided into two categories (≤ 0.5 μm and > 0.5 μm). Statistical significance p value was estimated using the Student’s t-test (two-tailed). Bar, 5 μm. (TIF) [file pgen.1009660.s002.tif]

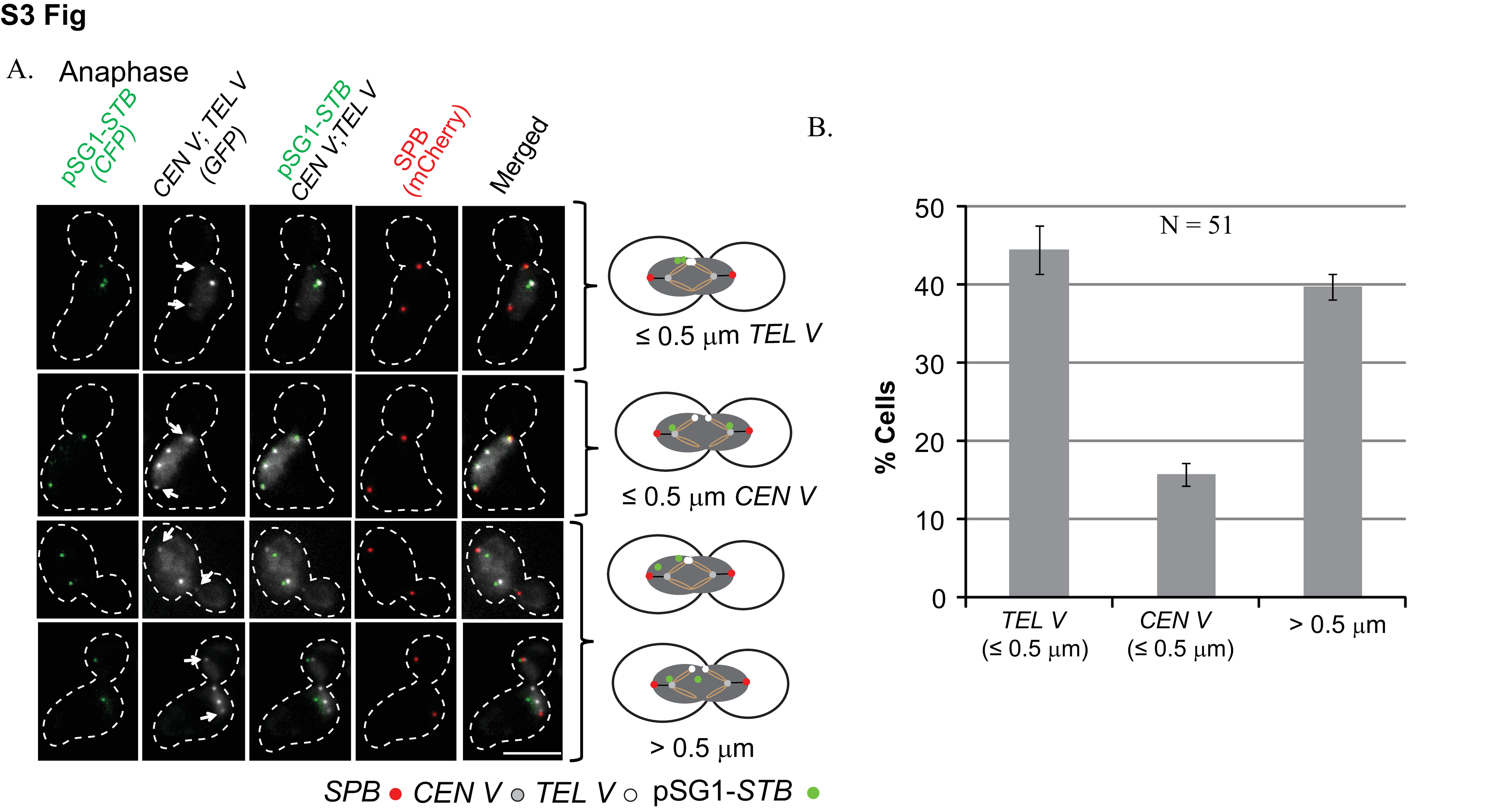

Supplement: S3 Fig — A. The representative images (rows at the left) depict a subset of early anaphase cells ([Cir+]; galactose grown) in which pSG1-STB was mapped with respect to well resolved CEN V (indicated by the arrows) but unresolved or closely spaced TEL V foci. Fluorescence tags: pSG1-STB, [LacO]256-[CFP-LacI]; CEN V, [TetO]224-[TetR-GFP]; TEL V, [TetO]448-[TetR-GFP]; SPB, (Spc42-mCherry). The pSG1-STB positions were classified into three types as idealized by the schematic diagrams at the right. B. The plot shows the relative frequencies of the three types. Placement in the ≤ 0.5 μm class required both plasmid copies to satisfy this criterion. The > 0.5 μm class included cells in which one or both of the plasmid copies exceeded the cut-off distance. Bar, 5 μm. (TIF) [file pgen.1009660.s003.tif]

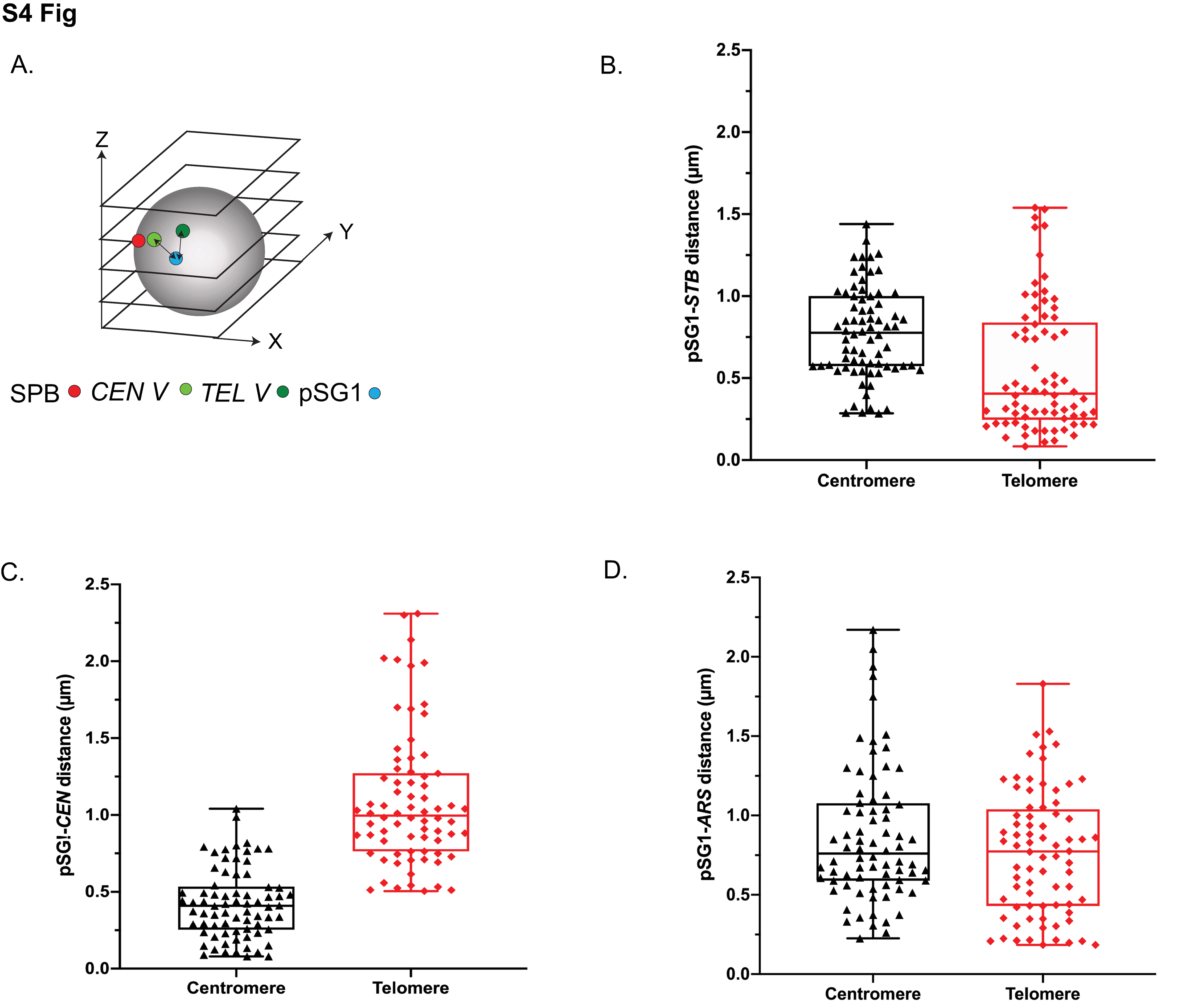

Supplement: S4 Fig — A. The positioning of the centroids of the fluorescent foci by Z-series sectioning of the nucleus is schematically shown. Images were captured from the [Cir0] experimental strain containing fluorescence-tagged SPB, CEN V and TEL V grown in glucose (pSG1-CEN) or galactose (pSG1-ARS) or the isogenic [Cir+] strain grown in galactose (pSG1-STB). Image analysis was performed using Imaris ‘Slice’ tool (see Materials and Methods for details). B-D. Plasmid distances from CEN V and TEL V are shown as dot plots. The data are based on scoring of at least 74 cells for each plasmid. (TIF) [file pgen.1009660.s004.tif]

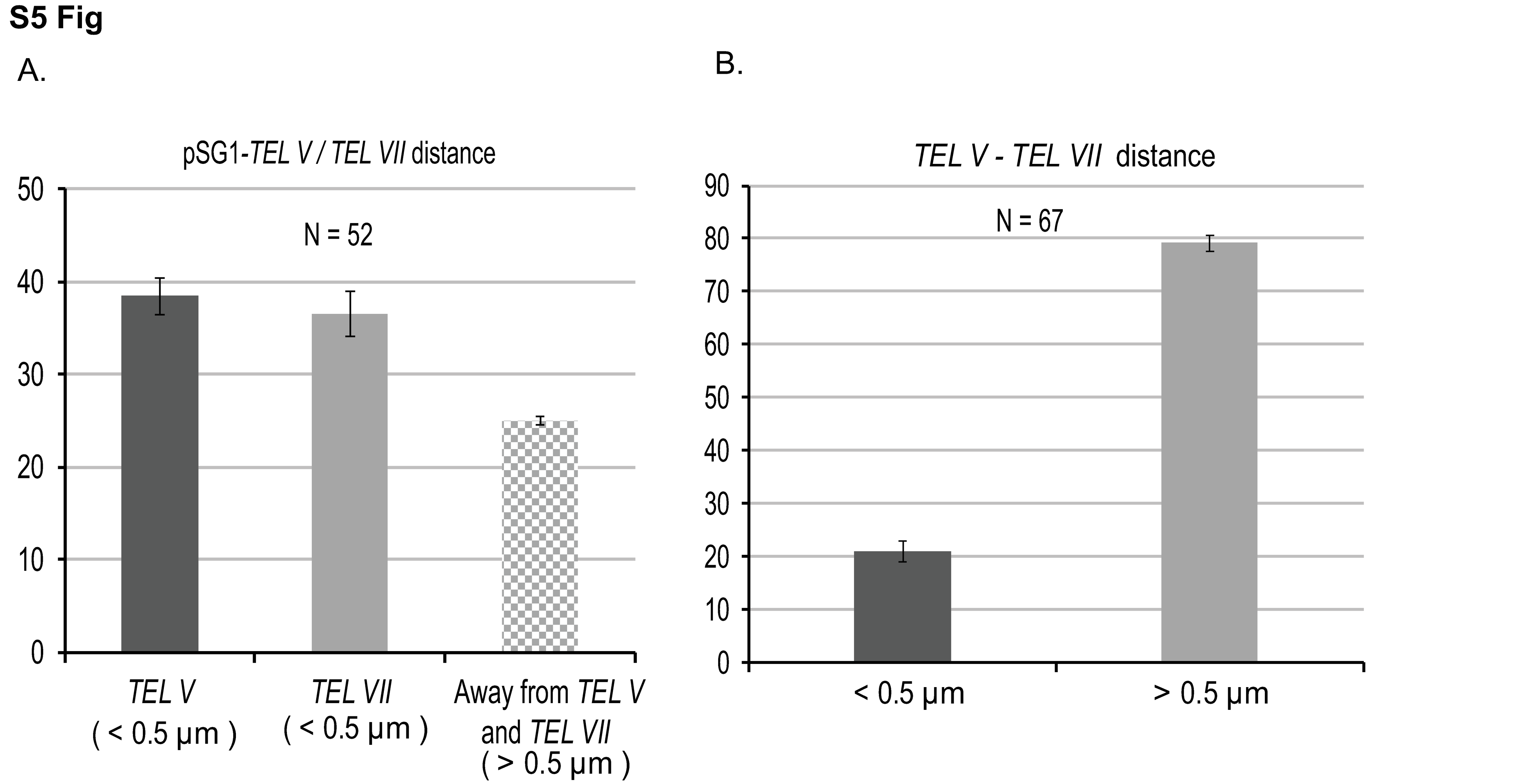

Supplement: S5 Fig — The analysis was similar to that described in the legend to Fig 2. The pSG1-STB plasmid ([Cir+]; galactose; [LacO]256-[CFP-LacI]) was localized with respect to TEL V and TEL VII in fixed G1/S cells. The TEL V and TEL VII tagged by [TetOn]-[TetR-GFP] were distinguished by the difference in the brightness of their fluorescence (TEL V >> TEL VII). A. The location of pSG1-STB was scored in the subset of cells in which TEL V and TEL VII were clearly resolved (>0.5 μm). B. The spacing of TEL V and TEL VII from each other was estimated. (TIF) [file pgen.1009660.s005.tif]

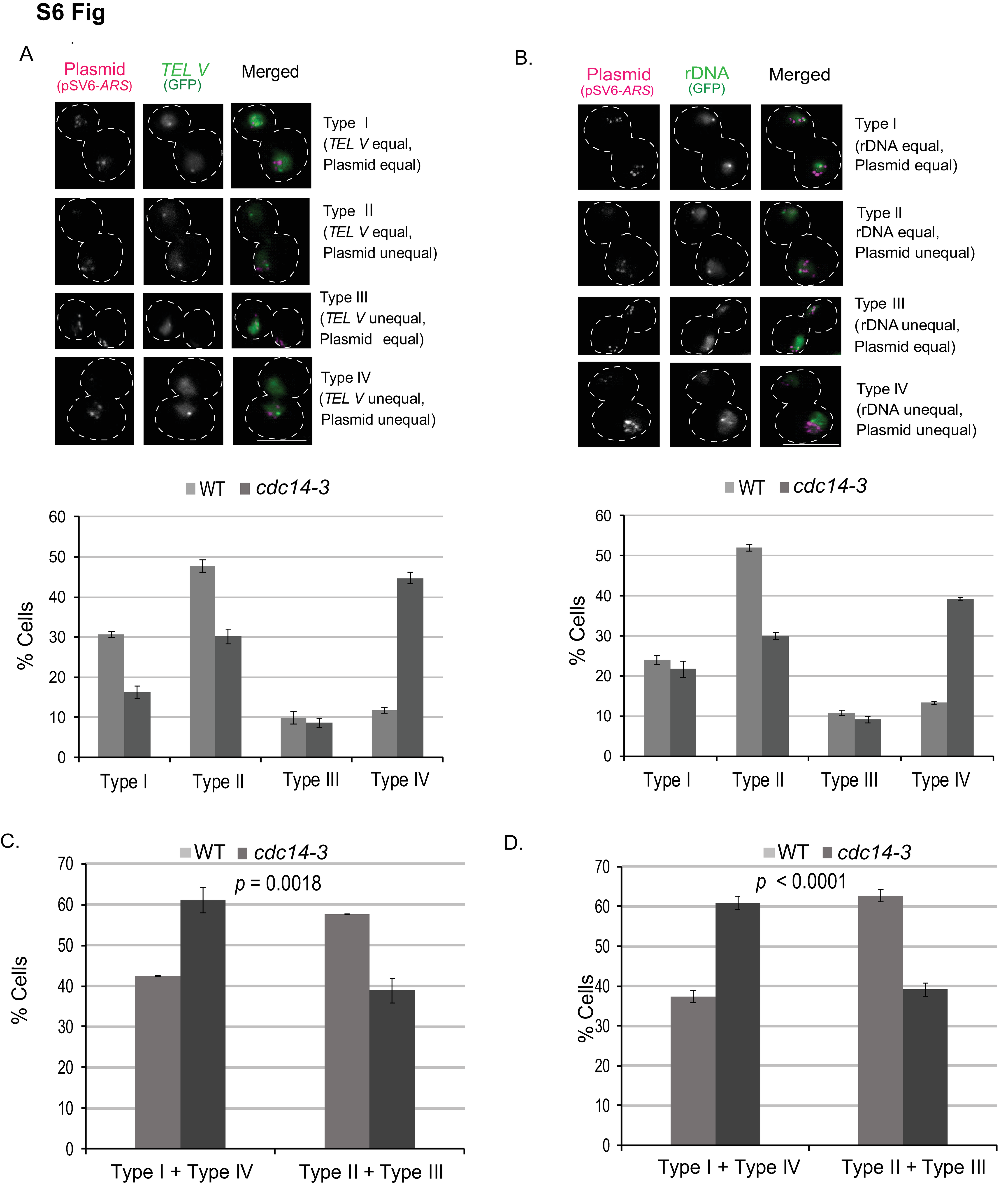

Supplement: S6 Fig — A, B. The experimental protocols were similar to those described under Fig 4, except that the reporter plasmid employed was pSV6-ARS. Plasmid segregation was assayed in conjunction with that of TEL V (A) or of rDNA (B). Fluorescence tags: pSV6-ARS, [LacO]256-[CFP-LacI]; TEL V, [TetO]448-[TetR-GFP]; rDNA, [TetO]448-[TetR-GFP]. The images of cell types (I-IV) and histogram plots of their quantitative analysis are arranged similarly to those in Fig 4. The (Type I + Type IV) and (Type II + Type III) plots represent plasmid segregation correlated and uncorrelated, respectively, with TEL V (C) and rDNA (D) segregation. The data for each assay set in wild type and cdc14-3 cells were derived from a minimum of 148 cells. Statistical analysis employed Fisher’s exact test. Bar, 5 μm. (TIF) [file pgen.1009660.s006.tif]

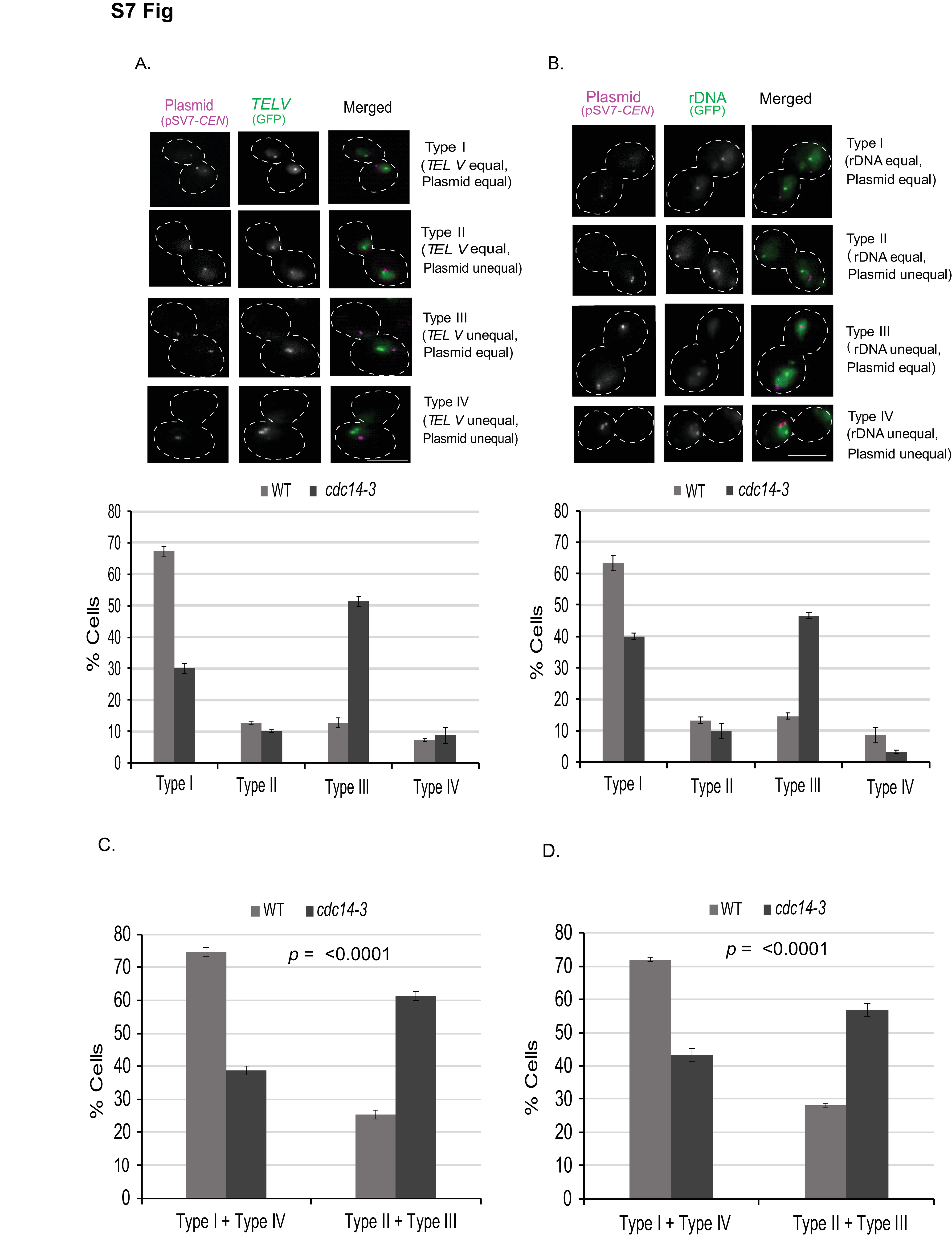

Supplement: S7 Fig — A, B. The assays in the wild type and cdc-14-3 strains (otherwise isogenic) were carried out as described in the legend to Fig 4 (also S6 Fig) with pSV7-CEN as the reporter plasmid. Fluorescence tags: pSV7-CEN, [LacO]256-[CFP-LacI]; TEL V, [TetO]448-[TetR-GFP]; rDNA, [TetO]448-[TetR-GFP]. The Types I-IV segregation represented by the cell images shown at the top are quantitated in the histogram plots below them. The (Type I + Type IV) and (Type II + Type III) plots represent plasmid segregation correlated and uncorrelated, respectively, with TEL V (C) and rDNA (D) segregation. At least 150 cells were scored in each set of assays for the wild type and cdc14-3 cells. Fisher’s exact test was used to estimate statistical significance. Bar, 5 μm. (TIF) [file pgen.1009660.s007.tif]

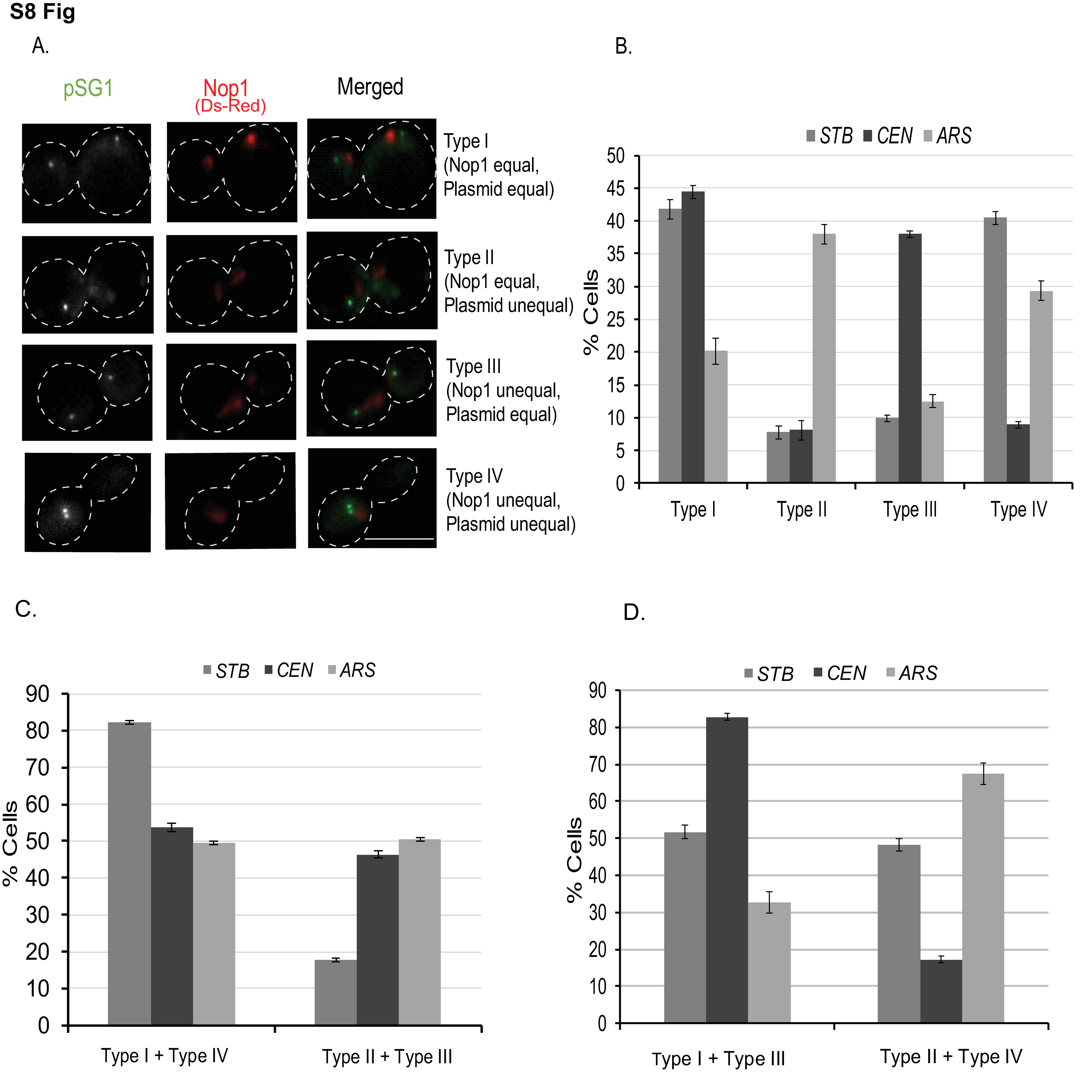

Supplement: S8 Fig — The partitioning status of the pSG1-reporter plasmid was manipulated as indicated in S1 Fig to obtain pSG1-STB, pSG1-CEN and pSG1-ARS. Segregation assays were performed at the non-permissive temperature (33°C). Fluorescence tags: pSG1, [LacO]256-[CFP-LacI]; Nop1 (DsRed). Segregation types I-IV represented in the cell images at the left (A) are quantitated in histogram plots at the right (B). The data shown in (C) and (D) are reformatted from those in (B). The plots in (C) highlight the distinctions between pSG1-STB and pSG1-CEN or pSG1-ARS in Nop1-correlated segregation (Type I + Type IV) and Nop1-uncorrelated segregation (Type II + Type III). In (D), overall plasmid segregation, Nop1-correlated or not, is displayed equal (Type I + Type III) or unequal (Type II + Type IV). For each plasmid type, over 200 cells were analyzed. Pairwise comparisons of pSG1-STB with pSG1-ARS or pSG1-CEN (C) by Fisher’s exact test showed statistical significance in each case (p < 0.0001). This was not the case for the pSG1-ARS and pSG1-CEN pair, p = 0.392. The Type I-Type IV distributions were statistically indistinguishable for the pSG1 plasmids (this figure) and the corresponding pSV set of plasmids (Figs 4, S6 and S7) under Cdc14 inactivation. For each pairwise comparison, chi-square test gave p > 0.05: pSV5-STB and pSG1-STB (p = 0.1350); pSV6-ARS and pSG1-ARS (p = 0.1618) and pSV7-CEN and pSG1-CEN (p = 0.0814). Bar, 5 μm. (TIF) [file pgen.1009660.s008.tif]

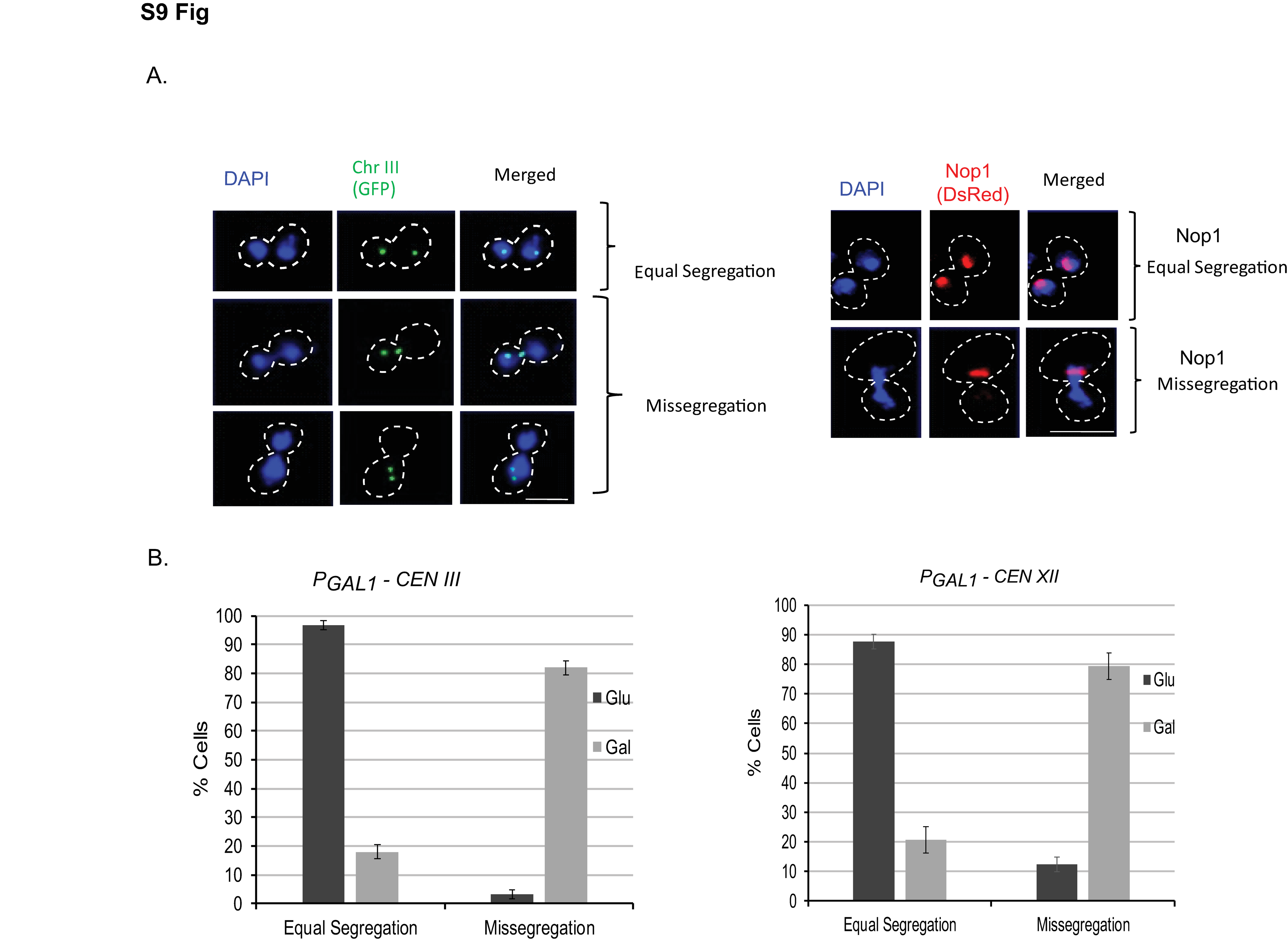

Supplement: S9 Fig — A. In the experimental strains, lacking a fluorescence-tagged reporter plasmid, the GAL1 promoter-controlled CEN III or CEN XII was kept active or inactive in glucose or galactose, respectively. The strain with the conditionally active CEN III was engineered to express GFP-LacI, and contained a [LacO]256 insertion within Chr III. The strain with the conditionally active CEN XII was engineered to express fluorescence-tagged Nop1 (DsRed). The images show equal segregation and missegregation of Chr III and Chr XII, as followed by green and red fluorescence, respectively. B. The data from the analysis of at least 120 cells for each assay are plotted. Bar, 5 μm. (TIF) [file pgen.1009660.s009.tif]

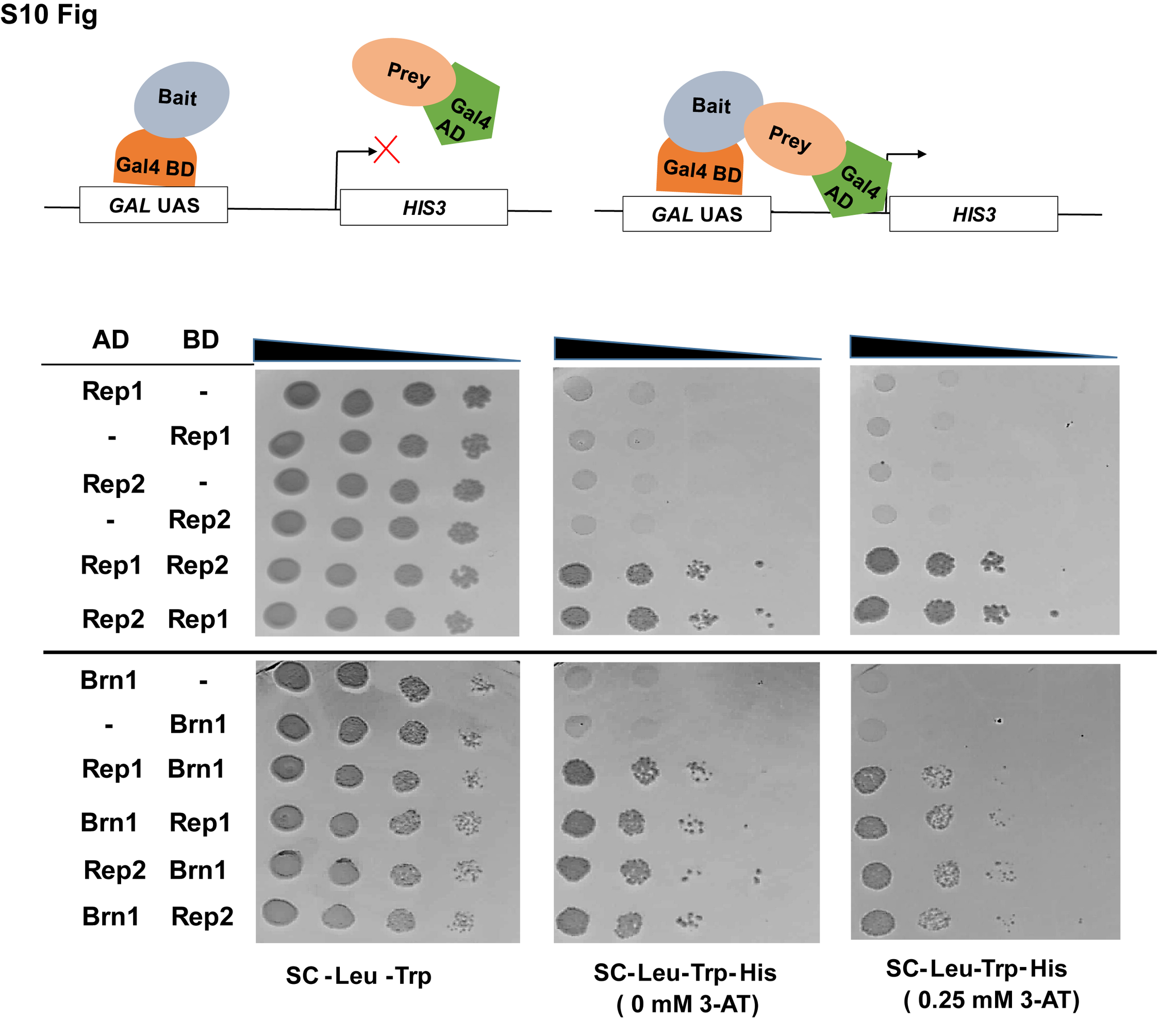

Supplement: S10 Fig — The dihybrid system based on the HIS3 reporter [108] is schematically diagrammed at the top (AD, activation domain; BD, binding domain). The expression vectors for ADH1 promoter-controlled AD- and BD-fusions were maintained by selection for the LEU2 and TRP1 markers, respectively, that they harbored. The indicated protein-protein interactions were assayed on SC-Leu-Trp-His and SC-Leu-Trp-His plates with or without added 3-AT. The interaction between Rep1 and Rep2 served as the positive control (last two rows of the top panel). The empty AD and BD vectors are indicated by ‘-‘. (TIF) [file pgen.1009660.s010.tif]
