# Supplementary material for: The selfish yeast plasmid utilizes the condensin complex and condensed chromatin for faithful partitioning
Source: PLoS Genet. 2021 Jul 16;17(7):e1009660. doi: 10.1371/journal.pgen.1009660 (PMC8318298; doi:10.1371/journal.pgen.1009660)
Supplement: S1 Table — The list of yeast strains and plasmids used in this study. (DOCX) [file pgen.1009660.s011.docx]

**Supporting Information**

**S1 Table. Strains and plasmids.**

The list of yeast strains and plasmids used in this study.

| **Strain Name** | **Genotype** | **Source / References** |
| --- | --- | --- |
| W303 CRY1[cir^+^] | *MAT* a *ade2-1 trp1-1 can1-100, leu2-3, 112 his3-11,15 ura3* [contains 2-micron plasmid] | Yeast Resource center (YRC) |
| W303 CRY1[cir^0^] | *MAT a ade2-1 trp1-1 can1-100, leu2-3, 112 his3-11,15 ura3* [lacks 2-micron plasmid] | Yeast Resource center (YRC |
| A5236 | *MAT* α ade2-1 trp1-1 can1-100 his3-11 prom*URA3::*TetR::GFP*::LEU2,telV::tetOX448::URA3* (between *BMH1* and *PDA1*) | [1] |
| A5238 | *MAT* α *cdc14-3* ade2-1 trp1-1 can1-100 his3-11 prom*URA3::*TetR::GFP*::LEU2,telV::tetOX448::URA3* (between *BMH1* and *PDA1*) | [1] |
| SGY3175 | *MAT* a ade2-1 trp1-1 can1-100 his3-11 prom*URA3::*TetR::GFP*::LEU2,telV::tetOX448::URA3* (between *BMH1* and *PDA1*) *CENV::tetOx224::HIS3, SPC42*-mCherry*::KanMx4,*[cir^+^] | This study |
| SGY3181 | *MAT* a ade2-1 trp1-1 can1-100 his3-11 prom*URA3::*TetR::GFP*::LEU2,telV::tetOX448::URA3* (between *BMH1* and *PDA1*) *CENV::tetOx224::HIS3, SPC42*-mCherry*::KanMx4,* CFP-LacI*::ADE2* [cir^+^] | This study |
| SGY3185 | *MAT* a ade2-1 trp1-1 can1-100 his3-11 prom*URA3::*TetR::GFP*::LEU2,telV::tetOX448::URA3* (between *BMH1* and *PDA1*) *CENV::tetOx224::HIS3, SPC42*-mCherry*::KanMx4,* CFP-LacI*::ADE2* [cir^0^] | This study |
| A9970 | *MAT* a *ade2-1 trp1-1 can1-100 his3-11* prom*URA3::*TetR::GFP*::LEU2,RDN1::tetOX112::URA3 [cir^+^]* | [1] |
| A9971 | *MAT* a *cdc14-3 ade2-1 trp1-1 can1-100 his3-11* prom*URA3::*TetR::GFP*::LEU2,RDN1::tetOX112::URA3 [cir^+^]* | [1] |
| SGY3218 | *MAT* a *ade2-1 trp1-1 can1-100 his3-11* prom*URA3::*TetR::GFP*::LEU2,RDN1::tetOX112::URA3,* CFP-LacI *::ADE2 [cir^+^]* | This study |
| SGY3219 | *MAT* a *cdc14-3 ade2-1 trp1-1 can1-100 his3-11* prom*URA3::*TetR*::*GFP*::LEU2,RDN1::tetOX112::URA3,* CFP-LacI *::ADE2 [cir^+^]* | This study |
| SGY3235 | *MAT* a *ade2-1 trp1-1 can1-100 his3-11* prom*URA3::*TetR::GFP*::LEU2,telV::tetOX448::URA3* (between *BMH1* and *PDA1*)*,* CFP-LacI:*:ADE2 [cir^+^]* | This study |
| SGY3236 | *MAT* a *cdc14-3 ade2-1 trp1-1 can1-100 his3-11* prom*URA3::*TetR::GFP*::LEU2,telV::tetOX448::URA3* (between *BMH1* and *PDA1*)*,* CFP-LacI*::ADE2 [cir^+^]* | This study |
| DY6280 | *MAT a ade2-1 can1-100 his3-11,15 leu2-3,112 lys2 met17 trp1-1 ura3-1 rad5-535 ,CEN3:: P_GAL1_v-CEN3-URA3 [cir^+^]* | [2] |
| DY6290 | *MAT a ade2-1 can1-100 his3-11,15 leu2-3,112 lys2 met17 trp1-1 ura3-1 rad5-535 ,CEN12:: P_GAL1_-CEN12-URA3 [cir^+^]* | [2] |
| SGY3239 | *MAT a 11,15 leu2-3,112 lys2 met17 trp1-1 ura3-1 rad5-535 ,CEN3:: P_GAL1_-CEN3-URA3,* GFP-LacI*:: ADE2, can1-100 his3- [cir^+^]* | This study |
| SGY3240 | *MAT a can1-100 his3-11,15 leu2-3,112 lys2 met17 trp1-1 ura3-1 rad5-535,CEN12:: P_GAL1_-CEN12-URA3,* GFP-LacI*:: ADE2, [cir^+^]* | This study |
| YIL-178-60 | *MAT* a *ade2-1, trp1-1 can1-100, leu2-3, 112 his3-11,15 ura3 , brn1-60 [cir^+^]* | [3] |
| SGY3095 | *MAT* a *trp1-1 can1-100, leu2-3, 112 his3-11,15 ura3,* GFP-LacI*:: ADE2 [cir^+^]* | This study |
| SGY3237 | *MAT* a*, trp1-1 can1-100, leu2-3, 112 his3-11,15 ura3, brn1-60* GFP-LacI*:: ADE2 [cir^+^]* | This study |
| SGY3222 | *MAT* a *ade2-1 trp1-1 can1-100, BRN1-6HA::HIS3* prom*URA3::*TetR::GFP*::LEU2,RDN1::tetOX112::URA3 [cir^+^]* | This study |
| SGY3223 | *MAT* a *cdc14-3 ade2-1 trp1-1 can1-100, BRN1-6HA::HIS3* prom*URA3::*TetR::GFP*::LEU2,RDN1::tetOX112::URA3 [cir^+^]* | This study |
| SGY3267 | *MAT* a *ade2-1 trp1-1 can1-100, BRN1-6HA::HIS3* prom*URA3::*TetR::GFP*::LEU2,RDN1::tetOX112::URA3 [cir^0^]* | This Study |
| SGY3268 | *MAT* a *cdc14-3 ade2-1 trp1-1 can1-100, BRN1-6HA::HIS3* prom*URA3::*TetR::GFP*::LEU2,RDN1::tetOX112::URA3 [cir^0^]* | This study |
| SGY3269 | *MAT a cdc14-1 ade2-1 trp1-1 ura3-1 can1-100 ,NOP1 RFP::LEU2,*TetR::YFP*::*CFP-LacI*::HIS3* | This study |
| PJ69-4A | *MATa trpl-901 leu2-3,112 ura3-52 his3-200ga14A ga18OA LYSZ::GALl-HIS3,GAL2-ADE2,metZ::GAL7-lacZ [*cir^+^*]* | [4] |
| CMY696 | *MAT α ade2-1 trp1-1 can1-100 his3-11, promURA3::TetR::GFP::LEU2, telV::tetOX448::URA3 (between BMH1 and PDA1), SPC42-mCherry::KanMx4, CFP-LacI::ADE2, telVII-TetOX(~50) (Between IMA1 and MAL13) [cir^+^]* | This Study |
| SGY11051 | *MAT a ade2-1, trp1-1 can1-100, leu2-3,15 ura3, BRN1-6HA::HIS3 [cir^0^]* | This Study |
| SGY11052 | *MAT a can1-100, leu2-3,15 ura3, BRN1-6HA::HIS3, promADH1::REP1::TRP1,pRS402-STB::ADE2[cir^0^]* | This Study |
| SGY11053 | *MAT a ade2-1, trp1-1 can1-100, leu2-3,15 ura3, BRN1-6HA::HIS3, promADH1::REP2::URA3,pRS402-STB::ADE2[cir^0^]* | This Study |
| SGY11054 | *MAT a trp1-1 can1-100, leu2-3, BRN1-6HA::HIS3, promADH1::REP1-REP2::URA3,pRS402-STB::ADE2[cir^0^]* | This Study |
| SGY11055 | *MAT a ade2-1, trp1-1 can1-100, leu2-3,15 ura3, BRN1-6HA::HIS3,pRS406::URA3,pRS402-STB::ADE2[cir^0^]* | This Study |
| SGY11056 | *MAT a trp1-1 can1-100, leu2-3,* *promADH1::REP1-REP2::URA3,pRS402-STB::ADE2 [cir^0^]* | This Study |
| **Plasmids** | **Salient features** | **Source / References** |
| pSV5 | Lac operator sequence (256 copies) cloned in YEpLac112 (*TRP1*) | [5] |
| pSG1 | P_GAL1_ -*CEN3-STB-ORI* cloned in pSV5 | [6] |
| pRS402-GFPLacI | GFP-LacI cloned in pRS402 plasmid (*ADE2*) | [7] |
| pRS402-CFPLacI | CFP-LacI cloned in pRS402 plasmid (after destruction of XhoI site after LacI) (*ADE2*) | This Study |
| pSV7-*CEN* | Lac operators (256 copies) cloned in YCpLac22 (*TRP1*) | [5] |
| pSV6-*ARS* | Lac operators (256 copies) cloned in YRp17 (*TRP1*) | [5] |
| pRS306-TetOX224 | TetO operators (224 copies) cloned in pRS306 plasmid | [8] |
| pRS424-TetO-*STB* | TetO operators (224 copies) cloned in pRS424 plasmid | This study |
| pUN100-dsRed Nop1 | The *CEN4* plasmid, expressing dsRed-Nop1 (*LEU2*) | [9] |
| pGADC1-*REP1* | *REP1* cloned in pGAD424 Vector for two-hybrid assay | This Study |
| GADC1-*REP2* | *REP2* cloned in pGAD424 Vector for two-hybrid assay | This Study |
| GADC1-*BRN1* | *BRN1* cloned in pGAD424 Vector for two-hybrid assay | This Study |
| GBDC1-*REP1* | *REP1* cloned in pGBDC1 vector for two-hybrid assay | This Study |
| GBDC1-*REP2* | *REP2* cloned in pGBDC1 vector for two-hybrid assay | This Study |
| GBDC1-*BRN1* | *BRN1* cloned in pGBDC1 Vector for two-hybrid assay | This Study |

**References**

1. D'Amours D, Stegmeier F, Amon A. Cdc14 and condensin control the dissolution of cohesin-independent chromosome linkages at repeated DNA. Cell. 2004;117(4):455-69. doi: 10.1016/s0092-8674(04)00413-1.

2. Reid RJ, Sunjevaric I, Voth WP, Ciccone S, Du W, Olsen AE, et al. Chromosome-scale genetic mapping using a set of 16 conditionally stable Saccharomyces cerevisiae chromosomes. Genetics. 2008;180(4):1799-808. doi: 10.1534/Fgenetics.108.087999.

3. Ouspenski II, Cabello OA, Brinkley B. Chromosome condensation factor Brn1p is required for chromatid separation in mitosis. Molecular Biology of the Cell. 2000;11(4):1305-13.

doi: 10.1091/mbc.11.4.1305.

4. James P, Halladay J, Craig EA. Genomic libraries and a host strain designed for highly efficient two-hybrid selection in yeast. Genetics. 1996;144(4):1425-36. Epub 1996/12/01. PubMed PMID: 8978031.

5. Mehta S, Yang XM, Chan CS, Dobson MJ, Jayaram M, Velmurugan S. The 2 micron plasmid purloins the yeast cohesin complex: a mechanism for coupling plasmid partitioning and chromosome segregation? The Journal of Cell Biology. 2002;158(4):625-37. doi: 10.1083/jcb.200204136.

6. Ghosh SK, Hajra S, Jayaram M. Faithful segregation of the multicopy yeast plasmid through cohesin-mediated recognition of sisters. Proceedings of the National Academy of Sciences. 2007;104(32):13034. doi: 10.1073/pnas.0702996104.

7. Hajra S, Ghosh SK, Jayaram M. The centromere-specific histone variant Cse4p (CENP-A) is essential for functional chromatin architecture at the yeast 2-microm circle partitioning locus and promotes equal plasmid segregation. The Journal of Cell Biology. 2006;174(6):779-90. doi: 10.1083/jcb.200603042.

8. Michaelis C, Ciosk R, Nasmyth K. Cohesins: chromosomal proteins that prevent premature separation of sister chromatids. Cell. 1997;91(1):35-45. doi: 10.1016/s0092-8674(01)80007-6.

9. Gadal O, Strauß D, Kessl J, Trumpower B, Tollervey D, Hurt E. Nuclear export of 60s ribosomal subunits depends on Xpo1p and requires a nuclear export sequence-containing factor, Nmd3p, that associates with the large subunit protein Rpl10p. Molecular and Cellular Biology. 2001;21(10):3405-15. doi: 10.1128/MCB.21.10.3405-3415.2001
