# Supplementary material for: The selfish yeast plasmid utilizes the condensin complex and condensed chromatin for faithful partitioning
Source: PLoS Genet. 2021 Jul 16;17(7):e1009660. doi: 10.1371/journal.pgen.1009660 (PMC8318298; doi:10.1371/journal.pgen.1009660)
Supplement: S2 Table — The DNA sequences of the primers used in Figs 7 and 8. (DOCX) [file pgen.1009660.s012.docx]

**Supporting Information**

**S2 Table. The list of primer sequences.**

The DNA sequences of the primers used in Figs. 7 and 8.

| **Locus Name** | **Forward Primer (5` to 3`)** | **Reverse Primer (5` to 3`)** |
| --- | --- | --- |
| ***CEN3*** | gatcagcgccaaacaatatgg | aacttccaccagtaaacgttt |
| ***STB*** | cgaagcatctgtgcttcattttgtagaaca | gaacaaaaaagaagtatagattctttgttg |
| ***TUB2*** | cttgtagacagcgtcatgg | cagatgtcataaagtgcttcg |
